# Single cell sequencing analysis identifies genetics-modulated *ORMDL3*^+^ cholangiocytes having higher metabolic effects on primary biliary cholangitis

**DOI:** 10.1186/s12951-021-01154-2

**Published:** 2021-12-06

**Authors:** Bingyu Xiang, Chunyu Deng, Fei Qiu, Jingjing Li, Shanshan Li, Huifang Zhang, Xiuli Lin, Yukuan Huang, Yijun Zhou, Jianzhong Su, Mingqin Lu, Yunlong Ma

**Affiliations:** 1 Department of Infectious Diseases, The First Affiliated Hospital of Wenzhou Medical University, Wenzhou, 325000 Zhejiang China; 2 School of Life Science and Technology, Harbin Institute of Technology, Harbin, 150080 China; 3 Institute of Biomedical Big Data, School of Ophthalmology and Optometry and Eye Hospital, School of Biomedical Engineering, Wenzhou Medical University, Wenzhou, 325027 Zhejiang China; 4 State Key Laboratory for Diagnosis and Treatment of Infectious Diseases, The First Affiliated Hospital, Collaborative Innovation Center for Diagnosis and Treatment of Infectious Diseases, Zhejiang University School of Medicine, Hangzhou, 310003 Zhejiang China; 5 Wenzhou Institute, University of Chinese Academy of Sciences, Wenzhou, 325011 Zhejiang China

**Keywords:** Single cell sequencing analysis, GWAS, Risk genes, PBC, ORMDL3, Liver cells

## Abstract

**Background:**

Primary biliary cholangitis (PBC) is a classical autoimmune disease, which is highly influenced by genetic determinants. Many genome-wide association studies (GWAS) have reported that numerous genetic loci were significantly associated with PBC susceptibility. However, the effects of genetic determinants on liver cells and its immune microenvironment for PBC remain unclear.

**Results:**

We constructed a powerful computational framework to integrate GWAS summary statistics with scRNA-seq data to uncover genetics-modulated liver cell subpopulations for PBC. Based on our multi-omics integrative analysis, 29 risk genes including *ORMDL3*, *GSNK2B*, and *DDAH2* were significantly associated with PBC susceptibility. By combining GWAS summary statistics with scRNA-seq data, we found that cholangiocytes exhibited a notable enrichment by PBC-related genetic association signals (Permuted P < 0.05). The risk gene of *ORMDL3* showed the highest expression proportion in cholangiocytes than other liver cells (22.38%). The *ORMDL3*^+^ cholangiocytes have prominently higher metabolism activity score than *ORMDL3*^−^ cholangiocytes (P = 1.38 × 10^–15^). Compared with *ORMDL3*^−^ cholangiocytes, there were 77 significantly differentially expressed genes among *ORMDL3*^+^ cholangiocytes (FDR < 0.05), and these significant genes were associated with autoimmune diseases-related functional terms or pathways. The *ORMDL3*^+^ cholangiocytes exhibited relatively high communications with macrophage and monocyte. Compared with *ORMDL3*^−^ cholangiocytes, the VEGF signaling pathway is specific for *ORMDL3*^+^ cholangiocytes to interact with other cell populations.

**Conclusions:**

To the best of our knowledge, this is the first study to integrate genetic information with single cell sequencing data for parsing genetics-influenced liver cells for PBC risk. We identified that *ORMDL3*^+^ cholangiocytes with higher metabolism activity play important immune-modulatory roles in the etiology of PBC.

**Graphical Abstract:**

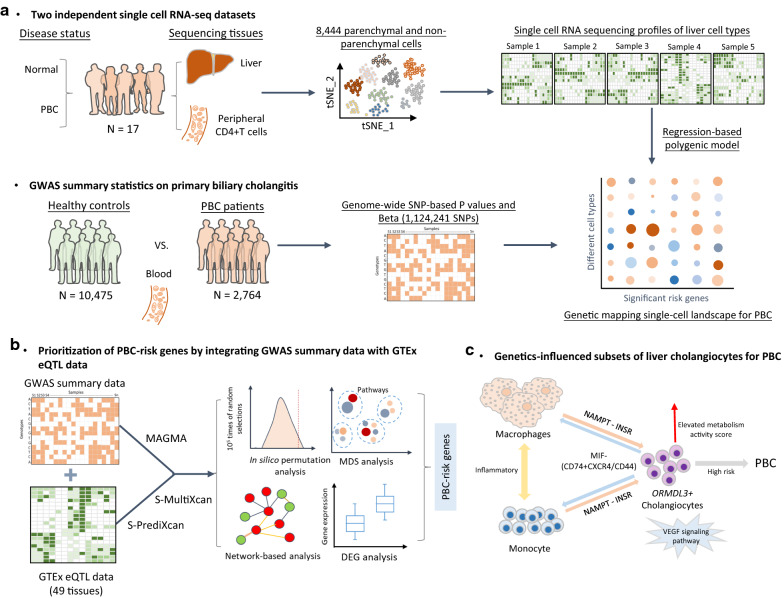

**Supplementary Information:**

The online version contains supplementary material available at 10.1186/s12951-021-01154-2.

## Background

Primary biliary cholangitis (PBC), which is formally known as primary biliary cirrhosis until 2016 [1], is a rare chronic cholestatic liver disease characterized by progressive autoimmune-mediated destruction of the small intrahepatic biliary epithelial cells [2, 3]. PBC patients suffering from chronic cholestasis can eventually lead to cirrhosis and hepatic failure without effective treatments [2]. Although ursodeoxycholic acid has been used as the first-line therapeutic agent for PBC, there exist 10–20% of PBC patients resistant to ursodeoxycholic acid and developing to advanced-stage liver disease [2]. Previous studies [4] have reported that a combination of genetic and environmental risk factors have an important influence on the aetiology of PBC. Hence, understanding the genetic mechanisms of PBC is becoming a great interest, which may promote the development of individualized therapeutic strategy for PBC.

Over the past decade, a growing number of genome-wide association studies (GWAS) and Immunochip studies based on East Asian and European populations have been performed to uncover the genetic susceptibility loci associated with PBC [4]. To date, more than 40 genetic loci with numerous risk genes have been reported [5–10], such as *SLC19A3/CCL20*, *IRF8/FOXF1*, *NFKB1/MANBA*, and *PDGFB/RPL3*. Nevertheless, the GWAS approach has generally focused on examining the genetic associations of millions of single nucleotide polymorphisms (SNPs) and only a handful of SNPs with a genome-wide significance (P ≤ 5 × 10^–8^) are reported. There exist many common SNPs with small marginal effects were neglected [11, 12]. Moreover, the vast majority of reported SNPs were mapped within non-coding genomic regions [12]. It is plausible to infer that these non-coding SNPs may modulate the expression levels of corresponding risk genes rather than change the functions of their proteins. Thus, combination of GWAS summary statistics and other different types of data that characterize tissue- and cell-type-specific activity, including expression quantitative trait loci (eQTL) [11], DNase I-hypersensitive sites (DHS) [13], and histone marks [14], contributes to highlight disease-related risk genes and cell types.

With the advance of single cell sequencing techniques, researchers have an effective avenue to discover more refined and novel cell populations for complex diseases [15]. An accruing and large number of single cell RNA sequencing (scRNA-seq) studies on autoimmune diseases, including rheumatoid arthritis [16], inflammatory bowel disease [17], and systemic lupus erythematosus [18], have been reported to parse the heterogeneity of cellular subpopulations at unprecedented resolution. In view of no scRNA-seq study was conducted for uncovering human liver cell types implicated in PBC, we constructed a computational framework to identify risk genes whose genetically expressions associated with PBC and pinpoint cell subpopulations implicated in the etiology of PBC.

## Results

### The framework for integrating single-cell transcriptomes and GWAS on PBC

**Fig. 1:**
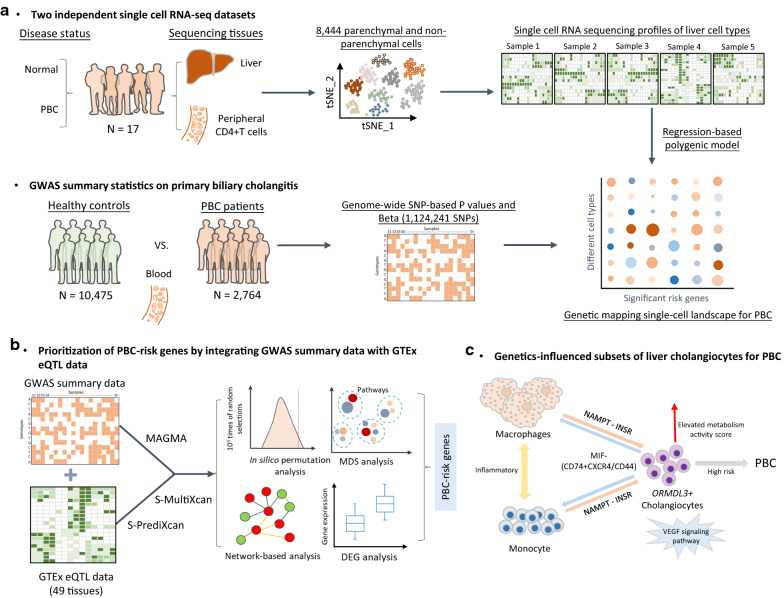
The workflow of current integrative genomics analysis. **a** Integrating single cell RNA-sequencing data with GWAS summary statistics on PBC based on a regression-based polygenic model; Left panel: Two independent single cell RNA-seq datasets based on liver and peripheral CD4 + T cells, and one large-scale GWAS summary statistics on PBC; Middle panel: There were 8444 parenchymal and non-parenchymal cells obtained the transcriptional profiles based on the CellRanger analysis pipeline and clustered by using t-Distributed Stochastic Neighbor Embedding (tSNE) method, and we also obtained 1,124,241 SNPs from the whole genome with P values and Beta value; Right panel: We applied a regression-based polygenic model in RolyPoly to unveil the genetic mapping single-cell landscape for PBC. **b** Prioritization of PBC-risk genes by integrating GWAS summary data with GTEx eQTL data; In this step, multiple bioinformatics analyses were leveraged to prioritize PBC-risk genes, including MAMGA-based gene-level association analysis, gene-property analysis, S-MultiXcan- and S-PrediXcan-based integrative genomics analyses, 10^5^ times of in silico permutation analysis, differential gene expression (DGE) analysis, multidimensional scaling (MDS) analysis, PPI network-based analysis, drug-gene interaction analysis, and functional enrichment analysis. **c** Genetics-influenced liver cell subpopulations and its immune microenvironment for PBC. We performed comprehensive single cell sequencing-based analyses to uncover the biological functions of *ORMDL3*^+^ cholangiocytes and its interacted immune cells of macrophages and monocytes. *ORMDL3*^+^ cholangiocytes have significantly elevated metabolism activity score, and VEGF signaling pathway has a crucial role in cellular communications of *ORMDL3*^+^ cholangiocytes

**Fig. 2:**
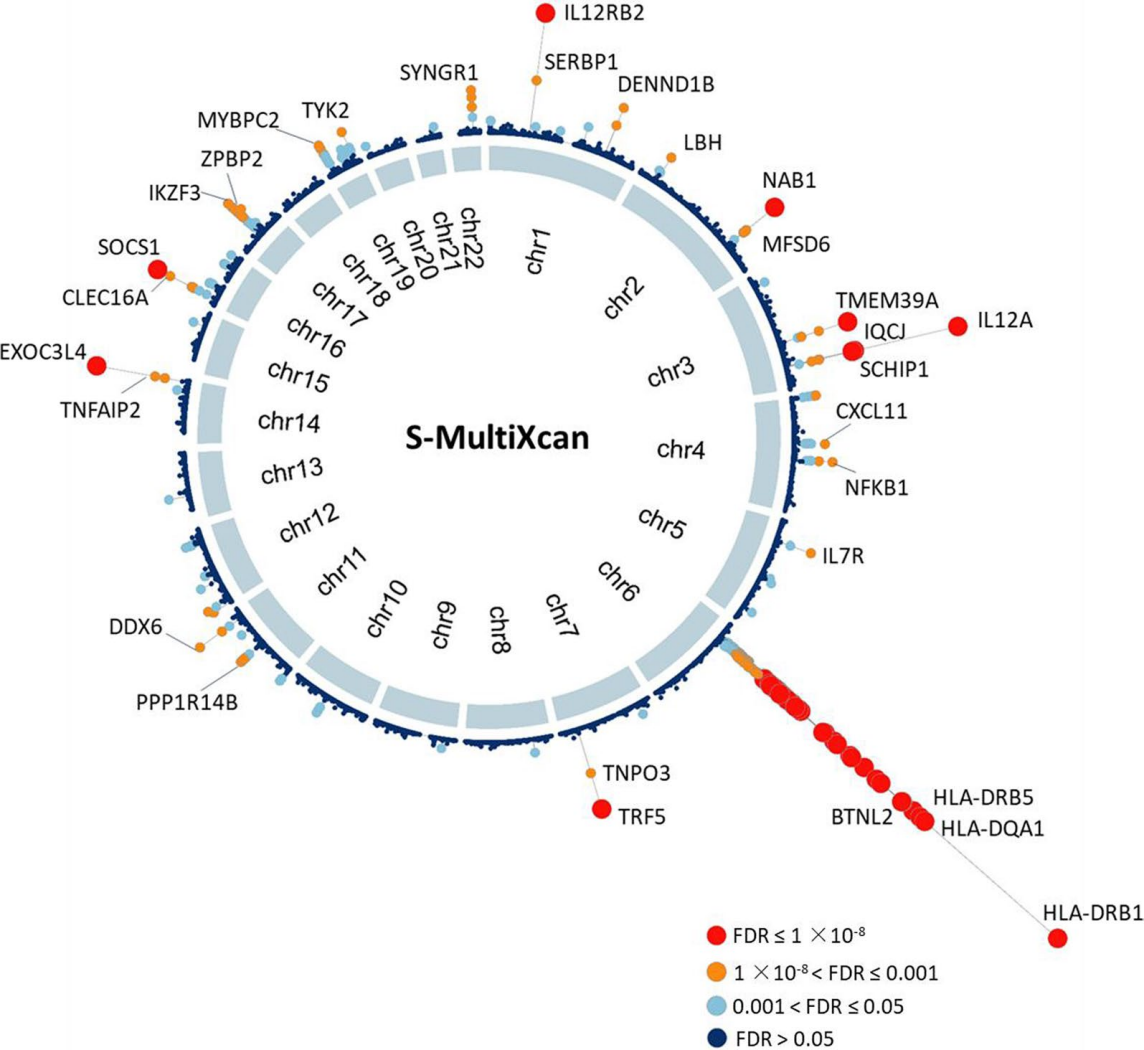
Circus plot showing the results of S-MultiXcan integrative genomics analysis. A circular symbol in the outer ring represents a given gene. Color represents the statistical significance of the gene, where red color marks significant genes with FDR ≤ 1 × 10^–8^, orange color marks significant genes with FDR is between 1 × 10^–8^ and 0.001, light blue indicates significant genes with FDR ranging from 0.001 to 0.05, and dark blue indicates non-significant genes with FDR > 0.05

**Fig. 3:**
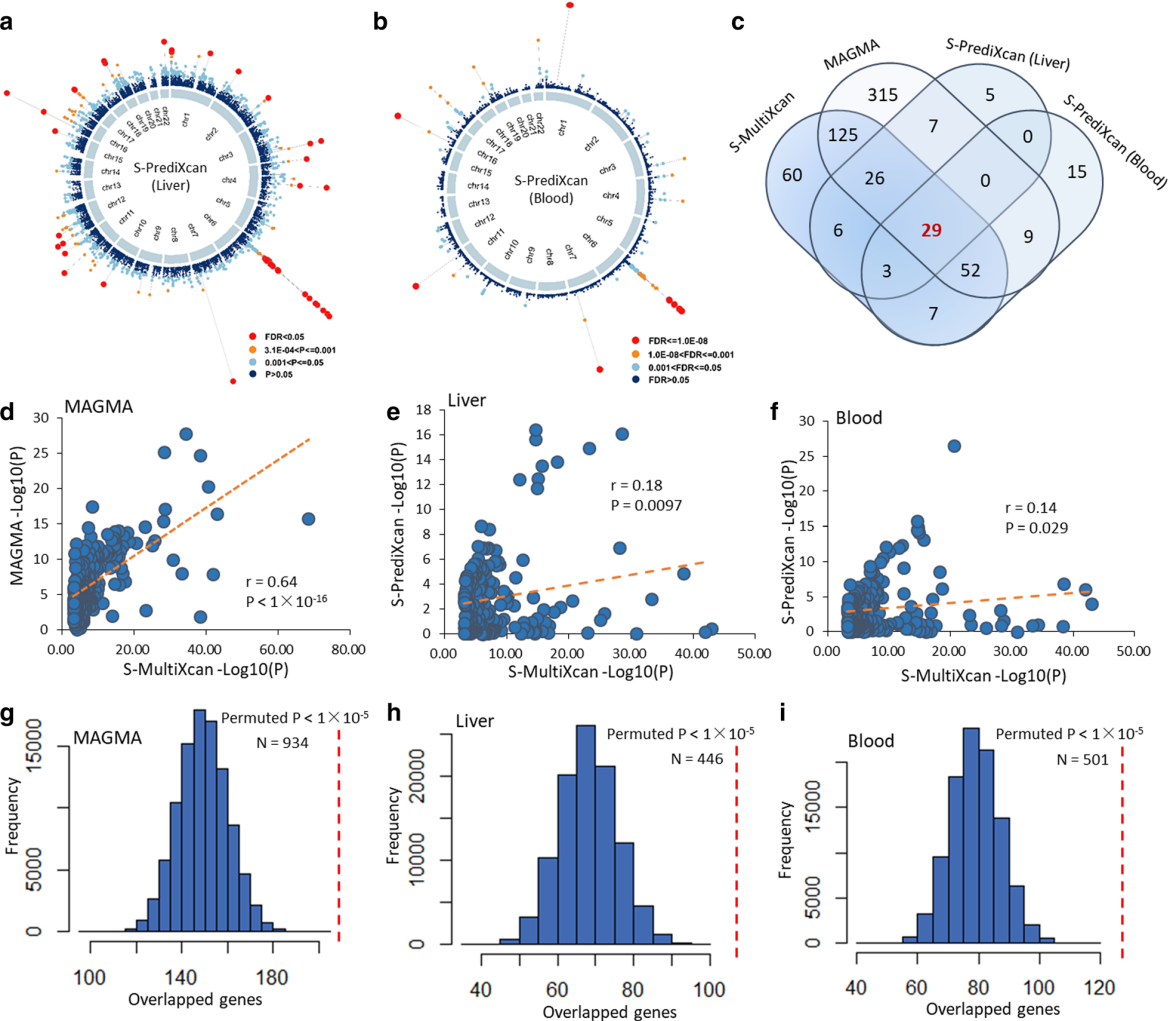
Results from integrative genomics analyses. **a**, **b** Circus plot demonstrates the results of S-PrediXcan integrative genomics analysis on (**a**) liver tissue and (**b**) blood tissue. **c** Venn plot showing the overlapped genes between S-MultiXcan, MAGMA, and S-PrediXcan on both liver and blood. **d**–**f** Pearson correlation of top-ranked genes identified from S-MultiXcan analysis with that from **d** MAGMA analysis, **e** S-PrediXcan analysis on liver, and **f** S-PrediXcan analysis on blood. **g**–**i** In silico permutation analysis of 100,000 random selections for the overlapped genes between S-MultiXcan and **g** MAGMA, **h** S-PrediXcan on liver, and **i** S-PrediXcan on blood

In the current study, we constructed a computational framework to unveil the cell-type specific genetic influence on the etiology of PBC based on multiple omics datasets (Fig. 1 and Additional file 2: Table S1). It included three primary sections: (1) combination of GWAS summary statistics on PBC with scRNA-seq data to recapitulate the genetics-modulated single cell landscape for PBC (Fig. 1a), (2) prioritization of genetics-risk genes and pathways contributing to PBC (Fig. 1b), and (3) revelation of the cell-to-cell interactions and metabolic activities of genetics-influenced subset of liver cholangiocytes and its immune microenvironment for PBC (Fig. 1c).

### Identification of gene-level genetic associations for PBC

To identify the aggregated effects of SNPs in a given gene on PBC, we performed a gene-level genetic association analysis and found that 563 genes were significantly associated with PBC (FDR ≤ 0.05, Additional file 1: Fig. S1, Additional file 2: Table S2). For example, the top-ranked genes of *HLA-DPA1* (P = 2.69 × 10^–24^), *IL12A* (P = 6.63 × 10^–22^), and *BTNL2* (P = 1.65 × 10^–17^). By using a genome-wide pathway analysis, there were 41 KEGG pathways significantly enriched (FDR ≤ 0.05, Additional file 2: Table S3 and Additional file 1: Fig. S2a). Based on the MDS analysis, these 41 pathways were grouped into five clusters, including Th1 and Th2 cell differentiation, allograft rejection, Th17 cell differentiation, cell adhesion molecules, and cytokine-cytokine receptor interactions (Additional file 1: Fig. S2b). The top-ranked pathways, such as Th1 and Th2 cell differentiation, allograft rejection, inflammatory bowel disease, and type I diabetes mellitus, were relevant to autoimmune diseases.

Furthermore, by performing a gene-property analysis based on mouse liver tissue with immune cells, we found that PBC-associated genes were significantly enriched in several immune-related cell types (Additional file 1: Fig. S3 and Additional file 2: Table S4), including Cst3^+^ dendritic cell (P = 5.8 × 10^–3^), Trbc2^+^ T cell (P = 7.1 × 10^–3^), Chil3^+^ macrophage (P = 0.017), and Gzma^+^ T cell (P = 0.03), which were in line with the results in previous studies [19, 20].

### Integrative analysis of GWAS summary statistics with eQTL data for PBC

To further highlight the functional genes whose expressions are associated with PBC, we leveraged the S-MultiXcan software [21] to meta-analyze tissue-specific associations across 49 GTEx tissues. There were 268 risk genes whose genetically-associated expression showing notable associations with PBC (FDR ≤ 0.05, Fig. 2). Among these significant genes, there were 52 risk genes having been documented in the GWAS Catalog database (Additional file 2: Table S5). Moreover, there was a high consistency of results between MAGMA and S-MultiXcan analysis (232/268 = 86.6%, Additional file 1: Fig. S4), such as *HLA-DRB1* (P = 4.95 × 10^–69^), *HLA-DRB5* (P = 1.17 × 10^–42^), and *BTNL2* (P = 3.26 × 10^–41^).

To further validate these risk genes in two PBC-relevant tissues (i.e., liver and blood) using the S-PrediXcan method, we found that 76 and 115 genes were significantly associated with PBC in liver and blood, respectively (FDR ≤ 0.05, Fig. 3a, b and Additional file 2: Tables S6, S7). In total, 29 risk genes were significantly associated with PBC across different methods including MAGMA, S-MultiXcan, and S-PrediXcan analyses (Fig. 3c). Using the Pearson correlation analysis, we observed that significant genes from S-MultiXcan analysis showed remarkable correlations with that from MAGMA and S-PrediXcan analyses (P ≤ 0.05, Fig. 3d–f). Moreover, by performing three independent permutation analyses, we found that the number of observed overlapped genes between S-MultiXcan and MAGMA and S-PrediXcan were significantly higher than random events (Empirical P < 1 × 10^–5^, Fig. 3g–i and Additional file 1: Fig. S5a–c). Overall, we identified 29 genes contribute susceptibility to PBC (Table 1).

**Table 1:** Identification of 29 significant risk genes by using integrative genomics analysis

Gene name	Z score	P value (S-MultiXcan)	FDR (S-MultiXcan)	FDR (S-PrediXcan on liver)	FDR (S-PrediXcan on blood)	FDR (MAGMA analysis of GWAS)	P value (Differential gene expression analysis)	GWAS_Catalog database or PubMed database
*CSNK2B*	1.40	7.17 × 10^–19^	8.41 × 10^–16^	3.22 × 10^–11^	2.24 × 10^–6^	1.67 × 10^–11^	4.96 × 10^–4^ (Blood), 2 × 10^–2^ (CD14 + T cell)	Novel gene
*LY6G5B*	− 7.92	2.43 × 10^–16^	1.93 × 10^–13^	5.52 × 10^–11^	1.54 × 10^–10^	1.19 × 10^–11^	Non-significant	Novel gene
*DDAH2*	− 5.57	1.39 × 10^–15^	9.99 × 10^–13^	2.65 × 10^–9^	1.56 × 10^–11^	1.69 × 10^–9^	2.70 × 10^–3^ (CD14 + T cell)	Novel gene
*LY6G5C*	− 7.82	2.40 × 10^–15^	1.62 × 10^–12^	8.64 × 10^–13^	9.05 × 10^–12^	1.41 × 10^–11^	2.80 × 10^–2^ (Blood)	Novel gene
*IRF5*	7.11	2.62 × 10^–15^	1.72 × 10^–12^	4.29 × 10^–13^	1.12 × 10^–12^	7.18 × 10^–11^	7.10 × 10^–3^ (Liver)	Reported gene
*SOCS1*	− 3.26	9.33 × 10^–13^	4.16 × 10^–10^	5.25 × 10^–10^	7.34 × 10^–10^	1.06 × 10^–8^	3.50 × 10^–2^ (CD14 + T cell)	Reported gene
*SYNGR1*	− 1.30	2.83 × 10^–9^	8.88 × 10^–7^	7.97 × 10^–4^	1.30 × 10^–7^	4.13 × 10^–2^	1.40 × 10^–2^ (CD14 + T cell)	Reported gene
*C6orf48*	− 4.46	9.57 × 10^–9^	2.66 × 10^–6^	7.83 × 10^–4^	6.19 × 10^–4^	4.83 × 10^–11^	4.16 × 10^–6^ (Blood)	Novel gene
*HLA-DMA*	− 3.99	8.87 × 10^–8^	1.90 × 10^–5^	1.27 × 10^–3^	9.16 × 10^–4^	1.62 × 10^–8^	2.10 × 10^–3^ (Liver)	Novel gene
*SMC4*	− 1.34	1.00 × 10^–7^	2.10 × 10^–5^	9.38 × 10^–5^	2.25 × 10^–6^	2.07 × 10^–4^	4.80 × 10^–4^ (Blood)	Novel gene
*TCF19*	4.55	1.49 × 10^–7^	2.99 × 10^–5^	9.46 × 10^–5^	2.24 × 10^–6^	1.98 × 10^–4^	Non-significant	Novel gene
*ORMDL3*	− 2.92	7.07 × 10^–7^	1.22 × 10^–4^	2.35 × 10^–3^	7.38 × 10^–5^	2.33 × 10^–6^	3.5 × 10^–2^ (Liver)	Reported gene
*KPNA4*	− 1.22	1.07 × 10^–6^	1.82 × 10^–4^	2.10 × 10^–2^	3.58 × 10^–3^	4.67 × 10^–4^	3.40 × 10^–3^ (CD14 + T cell)	Novel gene
*MANBA*	− 1.45	1.48 × 10^–6^	2.35 × 10^–4^	2.46 × 10^–6^	2.39 × 10^–2^	8.30 × 10^–8^	9.67 × 10^–5^ (Blood)	Reported gene
*MFSD6*	− 0.32	1.61 × 10^–6^	2.51 × 10^–4^	2.55 × 10^–2^	4.57 × 10^–2^	2.06 × 10^–3^	3.60 × 10^–2^ (Liver)	Novel gene
*MED1*	− 2.13	1.76 × 10^–6^	2.70 × 10^–4^	1.65 × 10^–2^	1.53 × 10^–2^	4.42 × 10^–5^	Non-significant	Novel gene
*IDUA*	3.95	2.37 × 10^–6^	3.47 × 10^–4^	3.94 × 10^–2^	3.87 × 10^–2^	8.95 × 10^–4^	8.10 × 10^–3^ (Blood)	Reported gene
*DGKQ*	3.24	3.86 × 10^–6^	5.37 × 10^–4^	4.90 × 10^–4^	3.36 × 10^–4^	2.35 × 10^–4^	8.34 × 10^–7^ (Blood)	Reported gene
*FCRL3*	− 4.40	8.14 × 10^–6^	1.07 × 10^–3^	2.84 × 10^–3^	2.84 × 10^–3^	5.81 × 10^–4^	Non-significant	Reported gene
*ZCRB1*	3.22	9.34 × 10^–6^	1.20 × 10^–3^	4.83 × 10^–3^	5.02 × 10^–3^	1.52 × 10^–3^	5.90 × 10^–3^ (Liver); 2.8 × 10^–3^ (CD14 + T cell);	Novel gene
*AGAP5*	− 4.28	1.29 × 10^–5^	1.59 × 10^–3^	4.50 × 10^–3^	3.40 × 10^–3^	3.65 × 10^–2^	Non-significant	Novel gene
*CARM1*	− 2.90	2.11 × 10^–5^	2.49 × 10^–3^	1.14 × 10^–3^	8.92 × 10^–4^	1.86 × 10^–4^	2.60 × 10^–2^ (Blood)	Novel gene
*UBE2D3*	− 4.18	2.83 × 10^–5^	3.23 × 10^–3^	2.76 × 10^–3^	5.08 × 10^–3^	5.81 × 10^–4^	3.40 × 10^–2^ (Blood); 1.6 × 10^–4^ (CD14 + T cell)	Novel gene
*NAAA*	4.08	6.73 × 10^–5^	6.88 × 10^–3^	1.25 × 10^–2^	6.85 × 10^–3^	7.41 × 10^–4^	3.20 × 10^–2^ (Liver)	Reported gene
*SH2B3*	− 1.69	1.20 × 10^–4^	1.17 × 10^–2^	2.35 × 10^–2^	1.87 × 10^–2^	2.44 × 10^–3^	1.40 × 10^–2^ (Blood)	Reported gene
*CTSH*	1.75	2.05 × 10^–4^	1.84 × 10^–2^	8.53 × 10^–3^	3.46 × 10^–2^	2.19 × 10^–5^	Non-significant	Novel gene
*TTC34*	3.56	2.82 × 10^–4^	2.39 × 10^–2^	5.86 × 10^–3^	5.02 × 10^–3^	4.98 × 10^–3^	9.80 × 10^–3^ (Blood)	Novel gene
*METTL1*	2.19	3.11 × 10^–4^	2.59 × 10^–2^	4.95 × 10^–2^	3.81 × 10^–2^	1.68 × 10^–2^	5.86 × 10^–7^ (Blood)	Novel gene
*TSFM*	3.45	3.14 × 10^–4^	2.61 × 10^–2^	4.87 × 10^–2^	3.87 × 10^–2^	1.54 × 10^–2^	2.80 × 10^–4^ (Blood); 1.5 × 10^–3^ (CD14 + T cell)	Novel gene

Based on three independent expression profiles of liver, blood, and peripheral CD4 + T cells, we conducted differential gene expression analysis for these 29 risk genes between PBC and matched control group. We found that 23 of 29 genes (79.31%) showed significantly differential expressions in PBC patients compared with controls (Table 1 and Additional file 1: Figures S6–S8). The co-expression patterns among these 29 genes in the peripheral CD4 + T cells were prominently altered according to the PBC status (Additional file 1: Fig. S8a). These results further support these identified risk genes have crucial effects on PBC.

### Functional analysis of these 29 PBC-associated risk genes

**Fig. 4:**
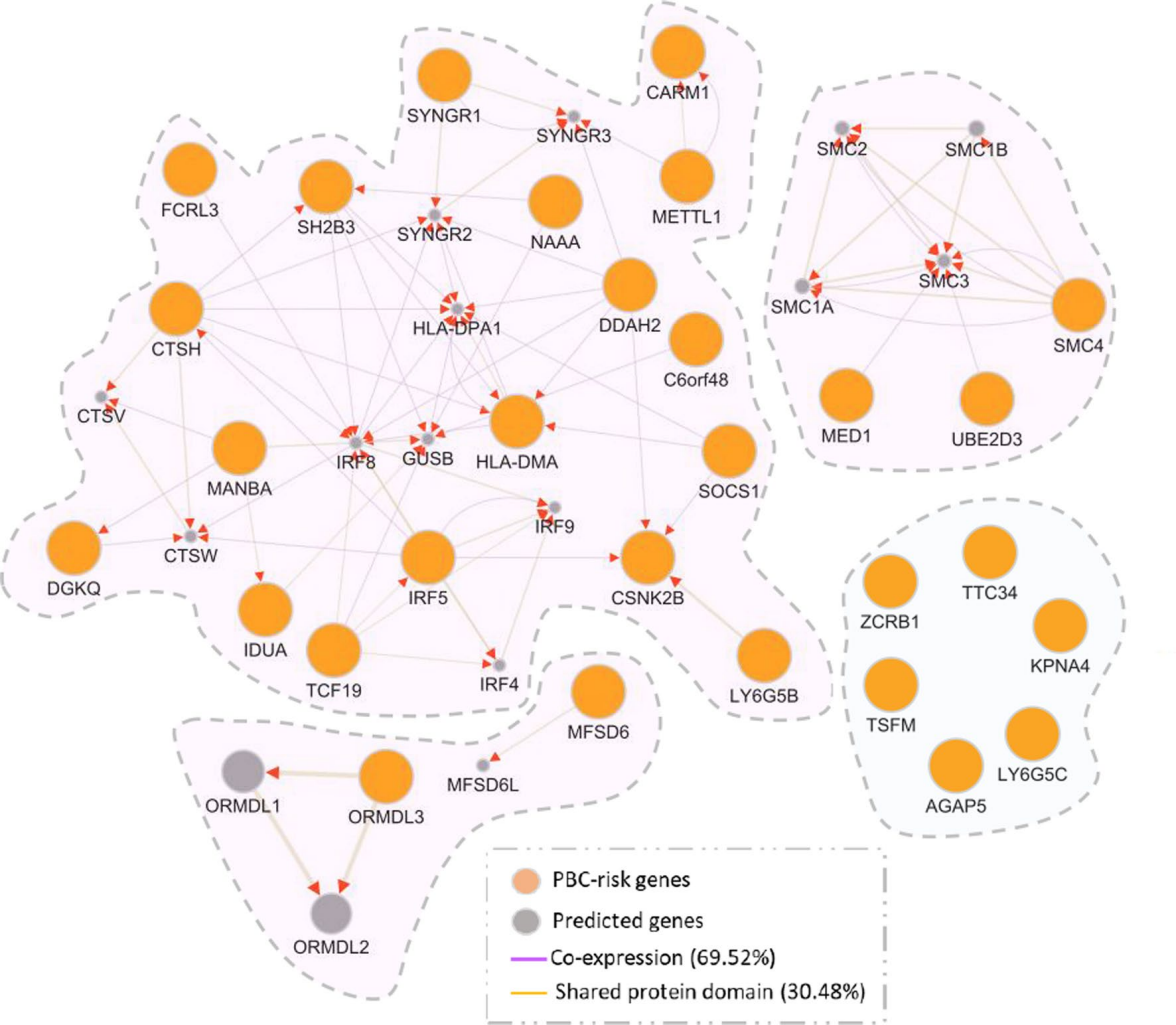
PPI network analysis of 29 PBC-risk gene. This network analysis was performed using the GeneMANIA tool. Orange node represents risk genes identified to be associated with PBC in our current analysis. Gray node represents predicted genes that connected with PBC-risk genes. Purple edge represents the co-expression interactions (account for 69.52%). Light orange edge represents the shared protein domain interactions (account for 30.48%)

Through mining the PubMed literature and GWAS Catalog database, we found that 10 of 29 identified risk genes have been reported to be associated with PBC in previous GWAS studies, and there were 19 novel genes newly identified (Table 1 and Additional file 1: Fig. S9a). Among these novel identified genes, several genes (e.g., *LY6G5B* and *DDAH2*) were associated with autoimmune-related diseases, including rheumatoid arthritis [22] and type 1 diabetes [23]. By conducting a PPI network enrichment analysis, we observed that these risk genes were significantly interacted with each other in a subnetwork (Fig. 4). For example, *TCF19* showed shared protein domains with *IRF5* [24], and *DDAH2* was highly co-expressed with *CSNK2B* [25].

Furthermore, we performed a phenotype-based enrichment analysis, and found that these 29 risk genes were remarkably enriched in several phenotypes relevant to autoimmune diseases (FDR ≤ 0.05, Additional file 1: Fig. S9b and Additional file 2: Table S8), such as type I diabetes mellitus, immune system diseases, and juvenile rheumatoid arthritis. We also performed a GO-term enrichment analysis, and found that several GO-terms were notably overrepresented (Additional file 1: Figs. S10–S12), such as interferon-gamma-mediated signaling pathway (P = 2.8 × 10^–4^) and neutrophil activation involved in immune response (P = 7.3 × 10^–4^).

Based on the drug-gene interaction analysis, we identified that 20 of 29 genes (68.96%) were enriched in ten potential “druggable” gene categories (Additional file 1: Fig. S13a and Additional file 2: Table S9). Five genes including *SOCS1, TCF19, CARM1, SH2B3,* and *NAAA* were directly targeted at least one known drug (Additional file 1: Fig. S13b). The gene of *SOCS1* was found to be targeted by insulin and aldesleukin, of which both have been applied to treat autoimmune diseases, including systemic lupus erythematosus [26], type 1 diabetes mellitus [27], and HIV [28]. *SH2B3* was targeted by ruxolitinib and candesartan. Previous studies [29] have demonstrated that the Janus kinase (JAK)-inhibitor ruxolitinib significantly influenced dendritic cell differentiation and function resulting in impaired T-cell activation, which could be used for the treatment of autoimmune diseases. These results provide a good drug repurposing resource to develop effective therapeutics for PBC.

### Identification of genetically-influenced liver cell subpopulations for PBC

**Fig. 5:**
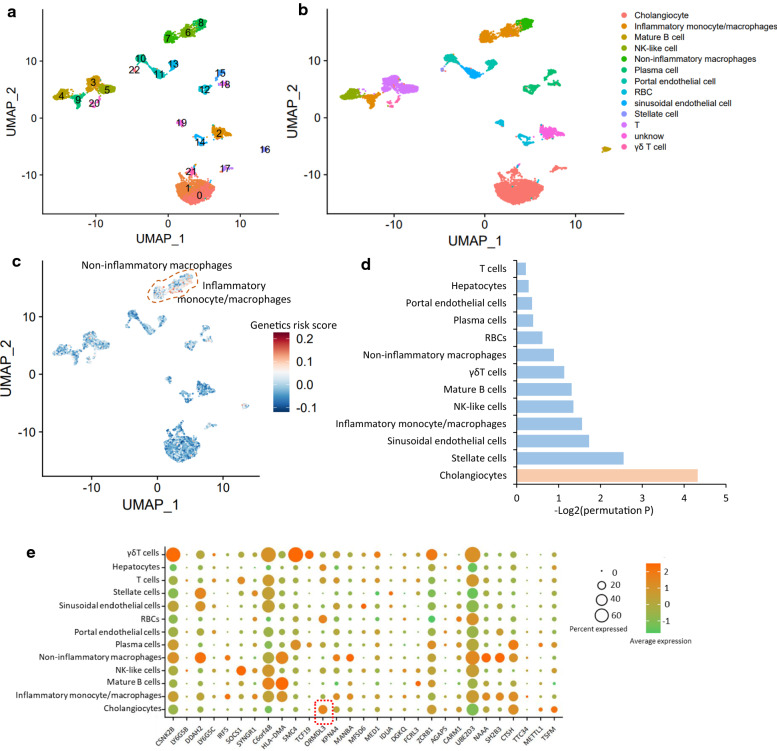
Genetics-influenced cell populations for PBC. **a** UMAP dimensionality reduction embedding of liver and immune cells among all five samples from primary liver patients (n = 8444 cells) colored by each cluster. **b** UMAP embedding of liver and immune cells colored by orthogonally generated clusters labeled by manual cell type annotation (13 cell types). **c** UMAP embedding of all cells among 13 cell populations colored by the genetic risk score of 29 PBC-risk genes. **d** Bar graph showing genetics-influenced liver cell subpopulations for PBC. Orange color represents PBC-relevant genetic association signals showing a significant enrichment in cholangiocytes. **e** Dot plot showing the expression percentage of 29 PBC-risk genes for each cell type from human liver tissue. The color stands for the average expression of each gene in each cell type, and the size of circular symbol indicates the percentage of a given gene expressed in each cell type

Using the uniform manifold approximation and projection (UMAP), there were 22 discrete clustered for the liver scRNA-seq dataset (Fig. 5a). Using well-known marker genes, these clusters were assigned into 13 distinct cell subpopulations, including portal endothelial cells, cholangiocytes, non-inflammatory macrophages, T cells, γδT cells, inflammatory monocytes/macrophages, natural killer (NK)-like cells, red blood cells (RBCs), sinusoidal endothelial cells, mature B cells, stellate cells, plasma cells, and hepatocytes (Fig. 5b). By calculating the genetic risk score of 29 PBC-associated genes using the *AddModuleScore* function in Seurat, we found that these risk genes were primarily enriched in non-inflammatory macrophage and inflammatory monocyte/macrophage (Fig. 5c), suggesting that these risk genes may have crucial functions in innate immunity for PBC. To uncover genetics-regulatory cell subpopulations associated with PBC, we used a regression-based polygenic model to combine GWAS summary statistics on PBC with scRNA-seq data with 13 distinct human liver cell types. We found that cholangiocytes showed a notable enrichment by PBC-relevant genetic association signals (Permuted P < 0.05, Fig. 5d and Additional file 2: Table S10). These results are consistent with previous evidence that an immune-mediated injury of cholangiocytes contributes risk to PBC [30, 31].

Using the specificity algorithm in the MAGMA method [32], we noticed that the risk gene of *ORMDL3* exhibited the highest expression in cholangiocytes than other cell types (Fig. 5e and Additional file 2: Table S11). The majority of *ORMDL3*-expressing cells were cholangiocytes with a relative high percentage of 22.38%, reminiscing that *ORMDL3* was demonstrated to be significantly associated with PBC in earlier studies [7, 9, 33]. Based on the liver expression profiles generated from healthy mice (n = 4) and mice suffering from cholangitis (n = 6) from the GEO database (GSE179993), we performed a differential gene expression analysis and found that the *ORMDL3* gene was significantly higher expressed among cholangitis-affected mice than that among healthy age- and sex-matched mice (P = 0.036, Additional file 1: Fig. S14). By further performing immunohistochemistry experiment, we observed that the expression of *ORMDL3* in liver tissues of PBC patients was higher than that in the normal liver, which is in line with the results from our genomics analysis (Additional file 1: Fig. S15). The top-ranked risk SNP associated with *ORMDL3* is rs9303277 (P = 2.57 × 10^–11^). This SNP showed significant cell-specific eQTL of *ORMDL3* among naïve B cell (P = 5.8 × 10^–10^), TFH^+^CD4^+^T cell (P = 3.8 × 10^–9^), CD56^dim^ CD16^+^ NK cells (P = 4.4 × 10^–9^), TH1^+^ CD4^+^ T cells (P = 6.0 × 10^–6^), and memory TREG^+^ CD4^+^ T cells (P = 1.1 × 10^–5^), and exhibited notable cell-specific promoter-interacting eQTL of *ORMDL3* among naïve B cell (P = 4.2 × 10^–13^) and CD56^dim^ CD16^+^ NK cells (P = 5.0 × 10^–12^) (Additional file 1: Fig. S16). Among these immune cell types, the CC genotype of rs9303277 shows prominent association with higher expression of *ORMDL3* compared with other genotypes (Additional file 1: Fig. S16a–k).

Growing attentions have concentrated on the role of *ORMDL3* in the development of inflammatory diseases including PBC [7, 9, 34]. In view of the main goal of current study was to characterize genetics-modulated liver cell subpopulations for PBC, the majority of our subsequent analyses focused on revealing the functions of *ORMDL3*^+^ cholangiocytes and its cellular communications with immune cells.

### Characterization of biological functions of *ORMDL3*^*+*^ cholangiocytes

**Fig. 6:**
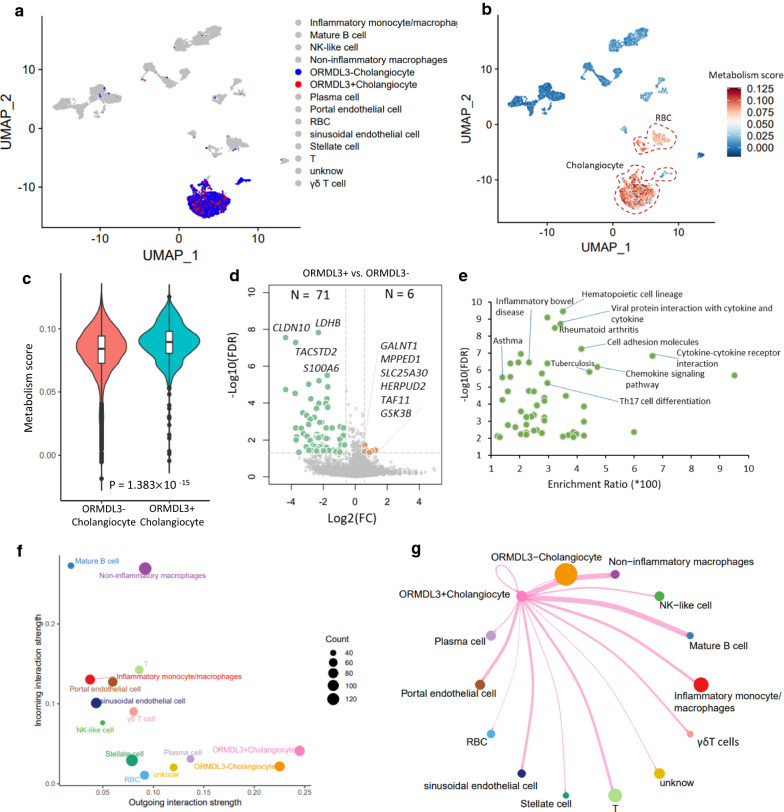
The biological functions of *ORMDL3*^+^ cholangiocytes. **a** UMAP projections of liver and immune cells colored by *ORMDL3*^+^ and *ORMDL3*^−^ cholangiocytes. Blue color represents *ORMDL3*^−^ cholangiocytes, red color represents *ORMDL3*^+^ cholangiocytes, and gray color stands for the rest of all other cell types. **b** UMAP embedding of all cells among 13 cell populations colored by the metabolism activity score of all metabolism-related pathways collected from the KEGG pathway resource. **c** Violin plot showing the difference in metabolism activity score between *ORMDL3*^+^ and *ORMDL3*^−^ cholangiocytes. Two-side Wilcoxon test was used for assessing significance. **d** Volcano plot showing the differentially expressed genes (DEGs) between *ORMDL3*^+^ cholangiocytes and *ORMDL3*^−^ cholangiocytes. Green color represents 71 significantly down-regulated DEGs, and orange color represents 6 significantly up-regulated DEGs. **e** Pathway enrichment analysis of 71 down-regulated DEGs based on the KEGG pathway resource. **f** Scatter plot showing the dominant senders (sources) and receivers (targets) in a 2D space. y axis represents incoming interaction strength, and x axis represents outgoing interaction strength. The size of each node represents the count of cellular interactions. **g** The cellular communications of *ORMDL3*^+^ Cholangiocyte with other cell populations in liver tissues. The width represents the number of cellular interactions

The subset of *ORMDL3*^+^ cholangiocytes account for 22.38% (635/2837) of cholangiocytes (Fig. 6a). By calculating the metabolism activity score among all annotated cells, we found that the cholangiocytes showed remarkably higher metabolism activity score than other cells (Fig. 6b). Interestingly, we noticed that the metabolism activity score of *ORMDL3*^+^ cholangiocytes was significantly higher than that in *ORMDL3*^*−*^ cholangiocytes (P = 1.383 × 10^–15^, Fig. 6c). Furthermore, we compared the expression profiles of *ORMDL3*^+^ cholangiocytes with *ORMDL3*^*−*^ cholangiocytes. There were 77 significantly DEGs with six up-regulated DEGs and 71 down-regulated DEGs among *ORMDL3*^+^ cholangiocytes (FDR < 0.05, Fig. 6d and Additional file 2: Tables S12–S13). With regard to six up-regulated DEGs, there were seven significant pathways overrepresented (FDR < 0.05, Additional file 1: Fig. S17a and Additional file 2: Table S14).

Among the 71 down-regulated DEGs, *HLA-DRA* has been shown to be associated with PBC [7], and *CXCL8*, *CCL3*, *CXCL1*, *TIMP1*, *SPP1*, and *IRF1* were inflammatory and cytokine genes, which have been reported to be linked with the chemotaxis of immune cells that efflux to the site of cytokine storms in response to ongoing tissue damage [35, 36]. *IFI16* is reported to be an innate immune sensor for intracellular DNA [37]. Pathway enrichment analysis revealed that there were 55 significant KEGG pathways enriched by these 71 down-regulated DEGs (FDR < 0.05, Fig. 6e and Additional file 2: Table S15), of which several such as inflammatory bowel disease, rheumatoid arthritis, Th17 cell differentiation, cytokine-cytokine receptor interaction, and chemokine signaling pathway have been demonstrated to implicate in inflammatory-related diseases [11, 38, 39].

To further explore the biological functions of these 71 down-regulated DEGs, we conducted GO-term enrichment analysis according to three categories of biological process (BP), cellular component (CC), and molecular function (MF). There were 19 BP-terms, 7 CC-terms, and 13 MF-terms showing notable enrichments, respectively (FDR < 0.05, Additional file 1: Fig. S17b–d and Additional file 2: Tables S16–S18). These functional terms were largely linked with immune and metabolism functions, such as granulocyte activation, neutrophil mediated immunity, and S100 protein binding. These results suggest that *ORMDL3*^+^ cholangiocytes have immune-modulatory effects on PBC risk.

### Cellular communications of *ORMDL3*^*+*^ cholangiocytes with other cells

**Fig. 7:**
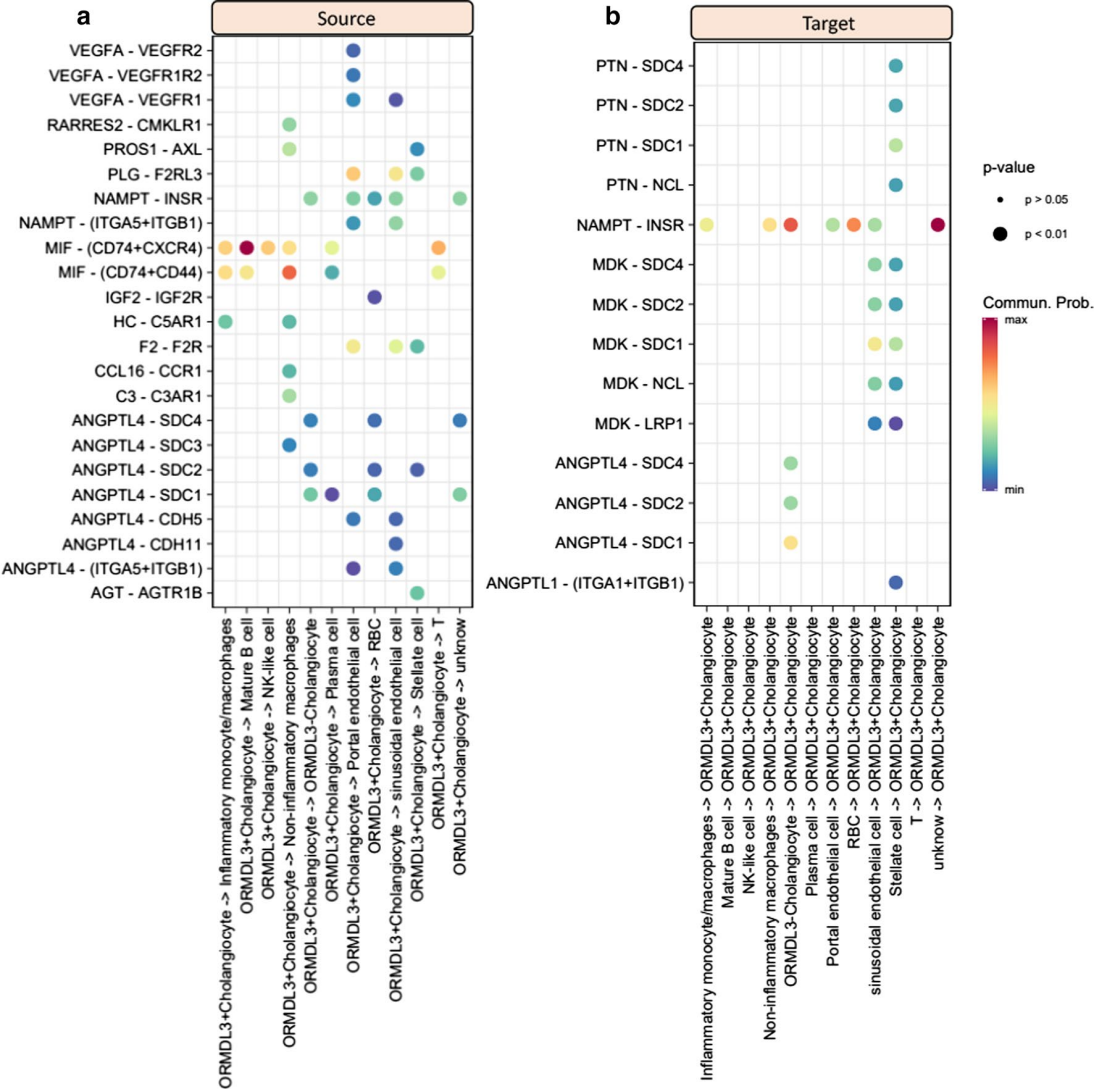
Dot plots showing that predicted cellular interactions of *ORMDL3*^+^ Cholangiocyte with other cell populations in liver tissues. **a** *ORMDL3*^+^ Cholangiocyte as a source (outgoing) communicating with other cells, **b** *ORMDL*^+^ Cholangiocyte as a target (incoming) communicating with other cells. The circular size of represents the statistical significance of each ligand-receptor pair, and color represents the communication probability

To gain refined insights into *ORMDL3*^+^ cholangiocytes, we performed a cell-to-cell interaction analysis among cell populations in liver tissue using the CellChat algorithm. By calculating the aggregated cell–cell communication network by counting the number of links, we found that *ORMDL3*^+^ cholangiocytes showed the highest outgoing (sources) interaction strength than other cell types (Fig. 6e and Additional file 1: Fig. S18). By further summarizing the communication probability among cellular interactions, we observed a high connective network of *ORMDL3*^+^ cholangiocytes with other cells (Fig. 6f and Additional file 1: Figs. S19, S20). The *ORMDL3*^+^ cholangiocytes showed relatively high communications with non-inflammatory macrophage and inflammatory monocyte/macrophages (Fig. 6f), recalling that these two types of immune cells have been remarkably enriched by PBC-risk genes in our above analysis. There were 35 significant ligand-receptor interactions (including source and target) of *ORMDL3*^+^ cholangiocytes were predicted, including MIF − (CD74 + CXCR4), MIF − (CD74 + CD44), VEGFA − VEGFR2, and NAMPT-INSR (Fig. 7a, b). There existed several unique ligand-receptor pairs in *ORMDL3*^+^ cholangiocytes compared with *ORMDL3*^*−*^ cholangiocytes, including VEGFA-VEGFR2, VEGFA-VEGFR1R2 and VEGFA-VEGFR1 (Additional file 1: Fig. S21).

**Fig. 8:**
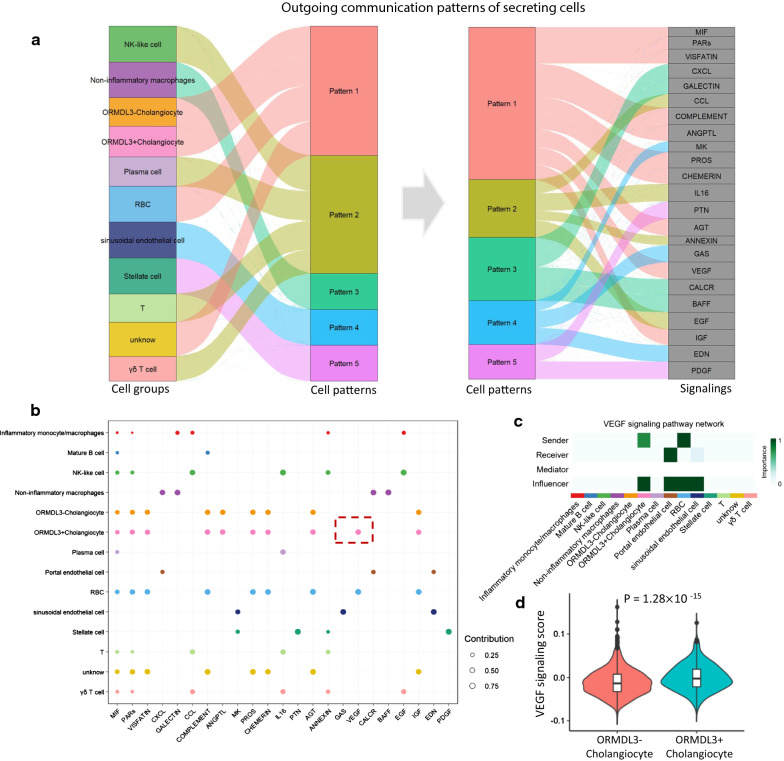
The inferred outgoing (source) communication patterns of cell populations in liver tissue. **a** Five inferred outgoing communications patterns of 13 cell populations, which shows the correspondence between the inferred latent patterns and cell groups, as well as signaling pathways, **b** The contribution of signaling pathways among the five outgoing communications patterns to liver and immune cell types. The red dashed box marked the VEGF signaling pathway. **c** Heatmap showing the relative importance of each cell group based on the computed four network centrality measures of VEGF signaling pathway network. **d** Violin plot showing the difference in VEGF signaling score between *ORMDL3*^+^ and *ORMDL3*^−^ cholangiocytes. The expression levels of all genes in VEGF signaling pathway were used to calculate the VEGF signaling score. Two-side Wilcoxon test was used for assessing significance

By performing a pattern recognition analysis based on the non-negative matrix factorization to uncover the global communication patterns among all cell populations, we observed that five communication patterns that connect cell populations with signaling pathways in the context of both outgoing signaling (i.e., treating cells as source, Fig. 8a) and incoming signaling (i.e., treating cells as target, Additional file 1: Fig. S22a), respectively. We found that a large portion of outgoing signaling of *ORMDL3*^+^ cholangiocytes is characterized by pattern #1, which represents multiple signaling pathways, including MIF, PARs, VISFATIN, COMPLEMENT, ANGPTL, PROS, CHEMERIN, AGT, VEGF, and IGF (Fig. 8b). Compared with *ORMDL3*^*−*^ cholangiocytes, the VEGF signaling pathway is specific for *ORMDL3*^+^ cholangiocytes as source cells to interact with other cells (Fig. 8b). On the other hand, the communication patterns of both *ORMDL3*^+^ and *ORMDL3*^*−*^ cholangiocytes as target cells showed similar patterns (Additional file 1: Fig. S22b). Network centrality analysis confirmed that VEGF signaling pathway is not only a significant sender (i.e., source cells) but also a prominent influencer controlling the communications for *ORMDL3*^+^ cholangiocytes (Fig. 8c). Notably, among all known ligand-receptor pairs, VEGF signaling pathway is dominated by VEGFA ligand and its VEGFR1 receptor (Additional file 1: Fig. S23). Using the expression levels of all genes in the VEGF signaling pathway to calculate a VEGF activity score, we found that *ORMDL3*^+^ cholangiocytes exhibited a significantly higher activity score than that among *ORMDL3*^*−*^ cholangiocytes (P = 1.28 × 10^–15^, Fig. 8d). These results suggest that VEGF signaling pathway play an important role in cellular communications of *ORMDL3*^+^ cholangiocytes.

## Discussion

Using an integrative genomics approach based on multiple layers of evidence, we identified that the genetics-influenced expression of 29 risk genes were remarkably associated with PBC. Among them, 10 genes, including *IRF5* [5, 6, 8, 9], *SOCS1* [5], *SYNGR1* [5, 7], *ORMDL3* [7, 9, 33], *MANBA* [5], *IDUA* [5], *DGKQ* [5], *FCRL3* [40], *NAAA* [41], and *SH2B3* [5], have been documented to be associated with PBC. There were 19 newly identified PBC-risk genes, such as *GSNK2B*, *LY6G5B*, *DDAH2*, *C6orf48*, and *HLA-DMA*. Some of these novel risk genes such as *DDAH2* and *HLA-DMA* have been reported to be associated with the autoimmune diseases, including type I diabetes [42], rheumatoid arthritis [43], and systemic lupus erythematosus [44]. The previously-reported gene of *ORMDL3* is related to biological functions of innate immune system and metabolism. Moreover, *ORMDL3* has also been extensively reported to be linked with other inflammatory diseases, including childhood asthma [45], inflammatory bowel diseases [38], rheumatoid arthritis [39], and Crohn’s disease [46]. Functional enrichment analyses uncovered that these genetics-risk genes were notably enriched in several biological processes or disease-terms, which are relevant to autoimmune phenotypes [47, 48]. Together, these identified genes are more likely to be genuine genes implicated in PBC risk.

Our gene-property analysis unveiled that PBC-relevant genetic association signals were significantly enriched in several immune-related cell populations, including dendritic cells, T cells, and macrophage. Consistently, based on the genetic risk score, we revealed that these 29 PBC-associated genes were prominently enriched in non-inflammatory macrophage and inflammatory monocyte/macrophage. Dendritic cells have been shown to be relevant to the pathogenesis of PBC [19, 49]. Earlier studies have demonstrated that an intense biliary inflammatory CD8 + and CD4 + T cell response has been used for characterizing PBC [50]. Moreover, dendritic cells are crucial for inducing antigen-specific T-cell tolerance and for activating the self-specific T cells, which have central roles in many aspects of the pathogenesis of autoimmune liver diseases [51]. Macrophages and monocytes are key components of the innate immune system, and have tissue-repairing roles in reducing immune responses and enhancing tissue regeneration [52]. Multiple lines of evidence [53] have demonstrated that the infiltration of macrophages and monocytes in diseased tissues is considered to be a hallmark of several autoimmune diseases, including PBC [20, 54]. Recently, Dorris et al. [55] reported that the PBC susceptibility gene *C5orf30* modulates macrophage-mediated immune regulations. Overall, these results suggest that genetics-risk genes have critical immuno-modulatory roles in the development of PBC.

To further examine the effects of liver cell populations and its immune microenvironments on PBC, we performed a regression-based polygenic analysis based on human liver tissues with immune cells. Cholangiocytes were significantly enriched by PBC-related genetic association signals. Previously, the non-parenchymal cholangiocytes have been reported to be injury in numerous human diseases termed as cholangiopathies [56], including PBC [56, 57]. Recently, Banales and coworkers [56] have demonstrated that cholangiocytes play pivotal roles in innate and adaptive immune responses relevant to immune-mediated cholangiopathies. Erice et al. [58] reported that microRNA-506 induces PBC-like features in human cholangiocytes and promotes the activating processes of immune responses. Moreover, we found that there existed higher metabolism activity score of cholangiocytes than other cells among liver tissues, indicating that abnormal metabolic pathways may involve in the etiology of PBC, which is in line with previous results [59]. Interestingly, the cell subset of *ORMDL3*^+^ cholangiocytes have strikingly higher metabolic activity than *ORMDL3* negative cells.

Compared with *ORMDL3*^*−*^ cholangiocytes, we found 77 significant DEGs among *ORMDL3*^+^ cholangiocytes, which contain numerous cytokine and chemokine genes, such as *CXCL8*, *CCL3*, and *CXCL1*, that may involve in mediating the immune-regulation for PBC risk. Furthermore, based on cellular communications analysis, we identified *ORMDL3*^+^ cholangiocytes exhibited high interactions with two innate immune cell types of non-inflammatory macrophage and inflammatory monocyte/macrophages. Several vital signaling pathways, including MIF, PARs, VEGF, and IGF, were predicted to implicate in the cellular interactions of *ORMDL3*^+^ cholangiocytes with other cells. The VEGF signaling pathway showed a higher specificity for *ORMDL3*^+^ cholangiocytes than *ORMDL3*^*−*^ cholangiocytes. VEGF is a potent stimulating factor for angiogenesis and vascular permeability, which have been reported to involve in PBC [60]. Multiple lines of evidence have demonstrated that VEGF has an important role in pathological conditions that are associated to autoimmune diseases, such as systemic lupus erythematosus, rheumatoid arthritis, inflammatory bowel disease, and multiple sclerosis [61].

There exist some limitations should be cautious. Although we leveraged integrated bioinformatics methods to highlight PBC-associated risk genes based on multiple omics data, there were many potential risk genes with suggestive evidence for PBC as shown in the supplemental tables needed to be further studied. Due to the heterogeneity across different datasets used in the present investigation, we leveraged different statistical methods for multiple testing correction for each dataset, such as FDR < 0.05 for MAGMA-based gene association analysis and S-MultiXcan analysis, permuted P < 0.05 for genome-wide pathway enrichment analysis, Bonferroni corrected P < 0.05 for gene-property analysis, and empirical P value < 0.05 for in silico permutation analysis. In view of current integrative genomic analysis is only based on samples derived from European ancestries, more studies based on other ancestries should be performed to validate the effects of these risk genes on PBC.

## Conclusions

In summary, current study provides multiple lines of evidence to support 29 genes including 19 novel genes are remarkably associated with PBC susceptibility. To the best of our knowledge, this is the first study to parse genetics-influenced human liver cell subpopulations and its immune microenvironments that contribute risk to PBC, and found that *ORMDL3*^+^ cholangiocytes potentially play important immune-regulatory roles in the pathogenesis of PBC. Current study gives several highlighted genetics-risk genes and disease-relevant cell types for unveiling the genetic mechanisms of PBC.

## Methods

### Datasets

1. *Single-cell transcriptomes of PBC* We downloaded two independent single cell RNA sequencing profiles (Accession number: GSE93170 and GSE115469) from the GEO database. With regard to the dataset of GSE93170, there were clinically and pathologically diagnosed six healthy controls and six PBC patients enrolled with written informed consent. Peripheral CD4 + T cells were used to extract total RNA. The Agilent microarray of SurePrint G3 human GE 8 × 60 K microarray kit was leveraged to produce gene expression profiles according to manufacturer’s protocols. The GSE115469 dataset contained five samples from primary liver patients, which were used for scRNA sequencing based on the 10 × Genomics Chromium Single Cell Kits. A total of 8444 parenchymal and non-parenchymal cells have obtained the transcriptional profiles based on the CellRanger analysis pipeline. The raw digital matrix of gene expression (namely UMI counts per gene per cell) was filtered, normalized and clustered. Cell was omitted if it has a very high (> 0.5) mitochondrial genome transcript ratio or a very small library size (< 1500).
2. *Bulk-based expression profiles of PBC* We also downloaded two independent bulk-based expression datasets based on liver tissue (Accession number: GSE159676) and blood (Accession number: GSE119600) from the Gene Expression Omnibus (GEO) database. The dataset of GSE159676 contained six healthy controls and three PBC cases based on fresh frozen liver tissue, which were obtained from explanted livers or diagnostic liver biopsies. The Affymetrix Human Gene 1.0-st array was leveraged to produce bulk-based liver expression profiles with 17,046 probes. With respect to the dataset of GSE119600, there were 47 healthy controls and 90 PBC patients with whole blood samples. The Illumina HumanHT-12 V4.0 expression beadchip was leveraged to produce bulk-based blood transcriptomes with 47,230 probes.
3. *GWAS summary statistics on PBC* We downloaded a GWAS summary dataset on PBC from the IEU open GWAS project (https://gwas.mrcieu.ac.uk/) [5]. There were 2764 PBC patients and 10,475 healthy controls based on European ancestry in this dataset used for performing a meta-analysis of GWAS signals. A standard quality control (QC) pipeline was applied to remove low-quality SNPs. The software package of MaCH [62] with the reference of HapMap3 CEU + TSI samples was implemented to perform a genome-wide imputation analysis. There were 1,124,241 SNPs with minor allele frequency > 0.005 and imputation quality score R^2^ > 0.5 included in the current analyses.

### Combination of GWAS summary statistics with scRNA-seq data for PBC

We leveraged a widely-used method of RolyRoly [63], which was designed to gain the effects of SNPs near protein-coding genes on cell types contributing to complex traits, to explore genetics-influenced liver cell types for PBC. The regression-based polygenic model was used in the RolyRoly to incorporate GWAS summary data with scRNA-seq data (i.e., GSE115469) for identifying PBC-associated liver cell subpopulations. Let $g(i)$ represents a given gene relevant to SNP $i$, $S_{j} = \{ i:g(i) = j\}$ represents a given set with multiple SNPs relevant to the gene $j$, and $\beta_{{S_{j} }}$ represents a PBC-GWAS-derived effect-size vector of $S_{j}$ with a *priori* assumption that $\beta_{{S_{j} }} \sim {\text{MVN}}\left( {0,\sigma_{j}^{2} I_{{|S_{j} |}} } \right)$. Under the assumption, RolyPoly offers a polygenic linear model for $\beta_{{S_{j} }}$:

$$\sigma_{j}^{2} = \gamma_{0} + \sum\limits_{i = 1}^{N} {\gamma_{i} \alpha_{ji} }$$

where $\gamma_{0}$ represents an intercept term, $\alpha_{ji} (i = 1,2,...,N)$ represents a group of annotations, for example, cell-type-specific gene expressions, and $\gamma_{i}$ is the annotation’s coefficient for $\alpha_{ji}$. To fit the observed and expected sum squared SNP effect sizes relevant to each gene, RolyPoly applies the method-of-moments estimators to estimate $\gamma_{i}$ by the following formula:

$$E\left( {\sum\limits_{{i \in S_{j} }} {\hat{\beta }_{i}^{2} } } \right) = \sigma_{j}^{2} {\text{Tr}}\left( {R_{{S_{j} }}^{2} } \right) + |S_{j} |\sigma_{e}^{2} n^{ - 1}$$

where $R_{{S_{j} }}$ represents the linkage disequilibrium (LD) matrix of $S_{j}$. The 1000 Genome Project European Phase 3 panel [64] was used to calculate the LD among SNPs. The major histocompatibility complex (MHC) region was removed due to the highly extensive LD in this region. RolyPoly utilized the 1000 iterations of block bootstrap to assess standard errors for calculating t-statistic and corresponding P value. P value ≤ 0.05 was interpreted to be of significance.

### Integrative genomic analysis of combining GWAS summary statistics with eQTL data

To highlight the functional risk genes whose expressions were significantly associated with PBC, we leveraged the S-PrediXcan tool [65] to combine GWAS summary data on PBC with eQTL data for liver and blood tissues from the GTEx Project (version 8) [66]. A MASHR-based prediction method with two linear regression models was used to estimate gene expression weights:

$$Y = \alpha_{1} + X_{l} \beta_{l} + \varepsilon_{1}$$

$$Y = \alpha_{2} + G_{g} \gamma_{g} + \varepsilon_{2}$$

where $\varepsilon_{1}$ and $\varepsilon_{2}$ are independent error terms, $\alpha_{1}$ and $\alpha_{2}$ are intercepts, $Y$ is the $n$ dimensional vector for $n$ samples, $X_{l}$ is the allelic dosage for SNP $l$ in n samples, $\beta_{l}$ is the effect size of SNP $l$, $G_{g} = \sum\nolimits_{i \in gene(g)} {\omega_{ig} X_{i} }$ is the predicted expression calculated by $\omega_{lg}$ and $X_{l}$, in which $\omega_{lg}$ is derived from the GTEx Project, and $\gamma_{g}$ is the effect size of $G_{g}$. The Wald-statistic Z score for each association is transformed as:

$$Z_{g} = \frac{{\hat{\gamma }_{g} }}{{se\left( {\hat{\gamma }_{g} } \right)}} \approx \sum\nolimits_{i \in gene(g)} {\omega_{ig} \frac{{\hat{\sigma }_{i} }}{{\hat{\sigma }_{g} }}\frac{{\hat{\beta }_{i} }}{{se\left( {\hat{\beta }_{i} } \right)}}}$$

where $\hat{\sigma }_{g}$ is the standard deviation of ${\text{G}}_{g}$ and can be calculated from the 1000 Genomes Project European Phase 3 Panel [64], $\hat{\beta }_{l}$ is the effect size from GWAS on PBC and $\hat{\sigma }_{l}$ is the standard deviation of $\hat{\beta }_{l}$.

To enhance the statistical power to identify significant genes whose expression has similar functions across different tissues, we leveraged the S-MultiXcan tool [21] to incorporate convergent evidence across 49 different GTEx tissues. The S-MultiXcan fits a multivariate regression model from multiple tissue models jointly:

$${\text{Y}} = \sum\limits_{j = 1}^{p} {{\text{T}}_{j} } g_{j} + {\text{e}} = {\text{Tg}} + {\text{e}}$$

where ${\tilde{\text{T}}}_{j} = \sum\nolimits_{i \in gene(j)} {\omega_{i} {\text{X}}_{i} }$ is the predicted expression of tissue $j$, and ${\text{T}}_{j}$ is the standardization of ${\tilde{\text{T}}}_{j}$ to $mean = 0$ and $standard deviation = 1$. $g_{j}$ is the effect size for the predicted gene expression in tissue $j$, ${\text{e}}$ is an error term with variance $\sigma_{e}^{2}$, and $p$ is the number of chosen tissues. There were 22,279 genes used in the multivariate regression model. P < 6.89 × 10^–4^ (FDR < 0.05) was considered to be significant.

### Gene-based genetic association analysis

We conducted a gene-level association analysis of the GWAS summary statistics on PBC by leveraging a multi-variant converging regression model in the Multi-marker Analysis of GenoMic Annotation (MAGMA) [32, 67]. The analyzed SNP set among each gene was depended on whether the SNP mapped into the body of the gene or its extended regions (± 20 kb downstream or upstream). The 1000 Genomes Project Phases 3 European Panel [64] was used to compute the LD between SNPs. The P value threshold was set to 0.0016, and the method of Benjamini–Hochberg false discovery rate (FDR) was applied for multiple testing correction [68]. Furthermore, to investigate the biological processes involved in PBC, we performed genome-wide pathway enrichment analysis using the *build-in* function of the MAGMA gene-set method [32]. The competitive P value of each pathway was computed by leveraging the results that the incorporated effect of genes in a given pathway are prominently larger than the incorporated effect of all rest genes. The *build-in* function of 10,000 permutations in MAGMA was used for adjusting competitive P values. We utilized the widely-used pathway resource of KEGG with latest version [69] for the MAGMA-based pathway enrichment analysis.

### Multidimensional scaling analysis

To obtain the similarity of enriched pathways identified from MAGMA-based pathway enrichment analysis, we carried out a multidimensional scaling (MDS) analysis to group these biological pathways. First, we arranged a *pathway.txt* file included all the significant enriched pathways, and then utilized the Jaccard distance algorithm [70] to calculate the pathway-pathway distance scores according to overlapped genes. Using the distance scores among pathways, we obtained the first two components of results from the MDS analysis (i.e., MDS1 and MDS2). Subsequently, we plotted the clusters of these identified pathways via MDS1 and MDS2 using the *symbols* function in *R* platform [71]. The most significant pathway (i.e., pathway has the lowest P value in each cluster) was used to mark each cluster.

### In silico* permutation *analysis

We referenced the method used in previous studies [11, 67, 68, 72] to perform an in silico permutation analysis of 100,000 times of random selections *(N*_*Total*_) for validating the consistency of results from S-MultiXcan analysis (Geneset #1, N = 308, FDR ≤ 0.05) with other results from three distinct analyses: MAGMA analysis (Geneset #2: N = 563, FDR ≤ 0.05), S-PrediXcan on liver (Geneset #3: N = 76, FDR ≤ 0.05), and S-PrediXcan on blood (Geneset #4: N = 115, FDR ≤ 0.05). First, we separately calculated the number of overlapped genes between Geneset #1 and Genesets #2, #3 and #4 (*N*_*Observation*_). Second, we used the total number of genes in MAGMA, S-PrediXcan on liver, and S-PrediXcan on blood as the background genes (*N*_*Background*_ = 18,068, 12,033, and 12,070, respectively). By randomly selecting the same number of genes as Genesets #2, #3, and #4 from the background genes to overlap with Geneset #1, we computed the number of overlapped genes in each time (*N*_*Random*_). The empirical P value was calculated by using the formula: P = $\frac{{N}_{Random} \ge {N}_{Observation}}{{N}_{Total}}$. The empirically permuted P value ≤ 0.05 is interpreted as significance. By using the same method as above, we also performed an in silico permutation analysis for results from S-MultiXcan, MAGMA, and S-PrediXcan (on liver and blood) at a significant level of P value ≤ 0.05 to further explore the consistency.

### Functional enrichment analysis of disease- and GO-terms

We conducted a disease-based enrichment analysis for these identified risk genes associated with PBC by using the WEB-based Gene SeT AnaLysis Toolkit (WebGestalt: http://www.webgestalt.org) [73] based on the GLAD4U database [74]. Moreover, we used the WebGestalt tool to perform a functional enrichment analysis for these common PBC-genetic risk genes based on the Gene Ontology (GO) database [75]. There were three categories of GO-terms: molecular function (MF), cellular component (CC), and biological process (BP). The number of genes in each GO-term is between 5 and 2000. The over-representation algorithm was adopted for evaluating the significant level for these enrichment analyses, and the Benjamini–Hochberg FDR method was used for multiple correction. In addition, the WebGestalt tool was used to conduct functional enrichment analyses for these significantly differentially expression genes (DEGs) between *ORMDL3*^+^ and *ORMDL3*^−^ cholangiocytes.

### Protein–protein interaction network analysis

To explore the functional interactions of these identified PBC-associated genes, we conducted a protein–protein interaction (PPI) network-based analysis by leveraging the GeneMANIA tool (http://www.genemania.org/), which is a widely-used *plug-in* of Cytoscape platform [76–78]. By using PBC-associated genes as an input list, the GeneMANIA would predict genes with similar biological functions and construct interacted links by incorporating current existing genomics and proteomics information, containing co-expression associations and shared protein domains.

### MAGMA gene-property analysis

To identify PBC-associated single cell subpopulations enriched by MAGMA-identified genes, we conducted MAGMA gene property analysis using the web-access tool of FUMA (https://fuma.ctglab.nl/) [79], which is a highly efficient and easy-to-use software, to integrate gene-level association signals from GWAS summary data on PBC [5] with scRNA-seq data based on liver tissue from the Mouse Cell Atlas [80]. There were 20 distinct cell types of liver tissue in the Mouse Cell Atlas. The Bonferroni method [81] was used for multiple testing correction.

### Immune cell-specific expression quantitative trait loci

To find the eQTL for PBC-risk SNPs, we first extracted genetic variants statistics around upstream and downstream 400 kb of the targeted genes from GWAS summary data. We used the *LocusZoom* (http://locuszoom.org/) to figure a regional association plot for each gene. Then, we explored the cis-eQTL and promotor-interacting eQTL (pieQTL) among the Database of Immune Cell Expression quantitative trait loci and Epigenomics (DICE, https://dice-database.org/landing) [82] for annotating functional genetic variants.

### Defining cell activity scores

We applied the cell activity score (CAS) to evaluate the immunological degree and metabolic activity of each cell type by using a pre-defined expression gene set [83]. The CASs were obtained by using a widely-used equation: CAS_*j*_ (*n*) = average [RLE (PGS_*j*_, *n*)] − average [RLE (CGS_*m*_, *n*)], where PGS_*j*_ is a pre-curated gene set *j* in a given cell *n,* and CGS_*m*_ is a control gene set that was randomly selected from aggregate expression levels bins, which gain a comparable distribution of expression levels and over size to that of the pre-defined gene set. RLE represents the relative expression of PGS_*j*_ or CGS_*m*_. The *AddModuleScore* function in the Seurat tool [84] was leveraged to calculate the CAS with default parameters. We used pre-collected 85 metabolism-related pathways (N = 1667 genes) in KEGG database [69], such as primary bile acid biosynthesis (ID: 00120), pyruvate metabolism (ID: 00620), and oxidative phosphorylation (ID: 00640), to define inflammatory score and metabolic activity score, respectively.

### Cellular interactions among different cell types

To unveil potential cell-to-cell communications of *ORMDL3*^+^ and *ORMDL3*^−^ cholangiocytes with other liver and immune cells, we leveraged the CellChat R package [85] to infer the cellular interactions based on the normalized scRNA-seq dataset (i.e., GSE115469). The algorithm of CellChat could examine the ligand-receptor interactions significance among different types of cells based on the expression of soluble agonist, soluble antagonist, and stimulatory and inhibitory membrane-bound co-receptors. By summing the probabilities of the ligand-receptor interactions among a given signaling pathway, we could calculate the communication probability for the pathway.

### Immunohistochemistry for the expression of ORMDL3 in liver

Through searching the information retrieval system, we found four patients diagnosed as PBC through pathology in the past 2 years in the First Affiliated Hospital of Wenzhou Medical University, and three of them were able to obtain liver tissue sections. Approved by Ethics Committee in Clinical Research (ECCR) of the First Affiliated Hospital of Wenzhou Medical University, the liver paraffin sections of the PBC patients and paracarcinoma paraffin section of intrahepatic metastasis of rectal stromal tumor patient as negative control were used in this research. Immunohistochemistry was performed to assess the differential expression of *ORMDL3* in liver between PBC patient and normal control. Briefly, 5-mm paraffin sections were deparaffinized with xylene and then rehydrated through descending grades of alcohol. Antigen retrieval was performed by heating heated (95 °C) in the 0.01 M sodium citrate buffer (pH 6.0) for 15 min and incubating in 3% hydrogen peroxide for 10 min to block endogenous peroxidases. The paraffin-embedded liver sections were incubated overnight at 4 °C with Anti-ORMDL3 antibody (Abcam, Cambridge, MA, USA), followed by the appropriate horseradish peroxidase-conjugated secondary antibodies (zsbio, Beijing, China). Diaminobenzidine (DAB) was used as the chromogenic substrate and the sections were observed under the microscopy.

### Statistical analysis

Differential gene expression (DGE) analyses between controls and PBC patients of three RNA expression datasets (i.e., GSE93170, GSE159676, and GSE119600) were examined by using the Student’s T-test. P value ≤ 0.05 was of significance. We also performed a co-expression pattern analysis in the dataset of GSE93170 for genetics-risk genes among PBC and healthy controls to evaluate whether the co-expression patterns were changed due to the disease status. The PLINK (v1.90) [86] was used to calculate the LD values among SNPs. The hypergeometric test was used to evaluate the significant enrichment for the disease- and GO-term enrichment analysis. The Jaccard distance algorithm [70] was used to assess the similarities among pathways.

## Supplementary Information

**Additional File 1: Figure S1.** Circus plot showing the results of gene-level genetic association analysis of GWAS summary statistics on PBC. **Figure S2.** MAGMA-based functional annotation analysis for GWAS summary data. **a**) MAGMA-based pathway enrichment analysis based on GWAS summary data on PBC. **b**) Multidimensional scaling analysis of 41 significant pathways identified MAGMA-based pathway analysis. **Figure S4.** Results of MAGMA gene-property analysis based on the web-access tool of FUMA. **Figure S2.** Venn plot shows the overlap of results between MAGMA and S-MultiXcan analysis. **Figure S5.** Permutation analysis of 100,000 times for results from MAGMA and S-PrediXcan (liver and blood) compared with results from S-MultiXcan across multiple tissues. **Figure S6.** Differential gene expression analysis of 29 risk genes based on bulk RNA profiles of liver tissue (Dataset of GSE159676). **Figure S7.** Differential gene expression analysis of 29 risk genes based on bulk RNA profiles of blood tissue (Dataset of GSE119600). **Figure S8.** Differential gene expression analysis of 29 risk genes based on bulk RNA profiles in CD4+T cells (Dataset of GSE93170). **Figure S9.** Functional characterization of 29 risk associated with PBC. **a**) Schematic diagram showing the literature mining and GWAS catalog searching for novel PBC-risk genes. **b**) Phenotype-based enrichment analysis based on the GLAD4U database for 29 risk genes. **Figure S10.** Functional enrichment analysis of GO-term of biological process for 29 PBC-risk genes. **Figure S11.** Functional enrichment analysis of GO-term of cellular components for 29 PBC-risk genes. **Figure S12.** Functional enrichment analysis of GO-term of molecular function for 29 PBC-risk genes. **Figure S13.** Gene-drug interaction analysis for these identified PBC-associated risk genes. **a**) Drug-gene interaction analysis showing 10 druggable gene categories. **b**) Drug-gene subnetwork. **Figure S14.** Violin plot showing the difference in the expression level of Ormdl3 between cholangitis-affected mice and healthy controls. **Figure S15.** The expression of ORMDL3 examined in PBC livers and paracarcinoma of intrahepatic metastasis by immunohistochemistry. **Figure S16.** Box plots showing the effects of different genotypes (CC, CT, and TT) of rs9303277 on the expression of ORMDL3 among different immune cell types. **Figure S17.** Functional enrichment analysis of differential expressed genes between ORMDL3+ and ORMDL3- cholangiocytes. **Figure S18.** Identify signals contributing most to outgoing (sources) or incoming (targets) signaling of cell populations in liver tissues (GSE115469). **Figure S19.** The aggregated cell-tocell communication network among cell populations in liver tissues (GSE115469). **Figure S20.** The cellular communications of ORMDL3- Cholangiocyte with other cell populations in liver tissues (GSE115469). **Figure S21.** Dot plots showing that predicted cellular interactions of ORMDL3- Cholangiocyte with other cell populations in liver tissues (GSE115469). **Figure S22.** The inferred incoming (target) communication patterns of cell populations in liver tissue (GSE115469). **Figure S23.** Relative contribution of each ligand-receptor pair to that of VEGF signaling pathway.

**Additional File 2: Table S1.** Summary of collected datasets in current integrative genomics analysis. **Table S2.** Significant genes identified from MAGMA-based gene association analysis (FDR < 0.05). **Table S3.** MAGMAidentified significant enriched pathways based on GWAS summary data. **Table S4.** MAGMA gene-property analysis identifies liver single cells based on the Mouse Cell Atlas. **Table S5.** Significant PBC-associated genes identified by the S-MultiXcan tool. **Table S6.** Significant genes identified from S-PrediXcan analysis by integrating GWAS summary data with GTEx liver eQTL data. **Table S7.** Significant genes identified from S-PrediXcan analysis by integrating GWAS summary data with GTEx blood eQTL data. **Table S8.** Phenotype-based enrichment analysis of these 29 identified risk genes for PBC based on the GLAD4U database. **Table S9.** PBC-associated risk genes matched in druggable gene categories. **Table S10.** Results of PBC-associated liver cell types by using Rolypolybased integrative analysis of combining GWAS summary data with scRNA-seq data. **Table S11.** The percentage of expressed 27 genetic risk genes in all 13 distinct cell types in human liver tissues. **Table S12.** The significantly upregulated DEGs among ORMDL3+ cholangiocytes. **Table S13.** The significantly down-regulated DEGs among ORMDL3+ cholangiocytes. **Table S14.** Significantly KEGG pathways enriched by up-regulated DEGs among ORMDL3+ cholangiocytes using the clusterProfiler tool. **Table S15.** Significantly KEGG pathways enriched by down-regulated DEGs among ORMDL3+ cholangiocytes using the clusterProfiler tool. **Table S16.** GO enrichment analysis according to biological process terms for 71 down-regulated DEGs among ORMDL3+ cholangiocytes. **Table S17.** GO enrichment analysis according to cellular component terms for 71 down-regulated DEGs among ORMDL3+ cholangiocytes. **Table S18.** GO enrichment analysis according to molecular function terms for 71 downregulated DEGs among ORMDL3+ cholangiocytes.

## Data Availability

All the GWAS summary data applied in the present analysis can be obtained from the IEU open GWAS project (https://gwas.mrcieu.ac.uk/). The GTEx eQTL data (version 8) can be gained from the Zenodo repository (https://zenodo.org/record/3518299#.Xv6Z6igzbgl). The single cell RNA sequencing data and bulk-based RNA expression data were downloaded from the NCBI GEO database (https://www.ncbi.nlm.nih.gov/gds/). All scripts used in the Methods are deposited in the public available GitHub repository: https://github.com/mayunlong89/PBC_project.

## Abbreviations

PBC: Primary biliary cholangitis
GWAS: Genome-wide association study
SNP: Single nucleotide polymorphism
eQTL: Expression quantitative trait loci
DHS: DNase I-hypersensitive site
scRNA-seq: Single cell RNA sequencing
GEO: The gene expression omnibus
QC: Quality control
LD: Linkage disequilibrium
MHC: Major histocompatibility complex
MAGMA: The Multi-marker Analysis of GenoMic Annotation
FDR: False discovery rate
MDS: Multidimensional scaling
WebGestalt: The WEB-based Gene SeT AnaLysis Toolkit
GO: Gene ontology
MF: Molecular function
BP: Biological process
CC: Cellular component
DEG: Differential expression genes
PPI: Protein–protein interaction
pieQTL: Promoter-interacting expression quantitative loci
DICE: The database of immune cell expression quantitative trait loci and epigenomics
CAS: Cell activity score
PGS: Pre-curated gene set
CGS: Control gene set
RLE: Relative expression
