# Supplementary material for: Single cell sequencing analysis identifies genetics-modulated ORMDL3+ cholangiocytes having higher metabolic effects on primary biliary cholangitis
Source: J Nanobiotechnology. 2021 Dec 6;19:406. doi: 10.1186/s12951-021-01154-2 (PMC8647381; doi:10.1186/s12951-021-01154-2)

**Additional Figures**

**Additional file Figure S1. Circus plot showing the results of gene-level genetic association analysis of GWAS summary statistics on PBC.** Note: This analysis was performed using the MAGMA software. A circular symbol in the outer ring stands for a given gene, and color of a circular symbol represents the statistical significance of a specific gene, i.e., red color marks genes significantly associated with PBC with FDR ≤ 1×10^-8^, orange color marks genes significantly associated with PBC with FDR ranging from 1×10^-8^ to 0.001, light blue indicates genes significantly associated with PBC with FDR ranging from 0.001 to 0.05, and dark blue indicates genes showing non-significant associations with PBC (FDR > 0.05). There were 563 genes showing significant associations with PBC (FDR ≤ 0.05).

**
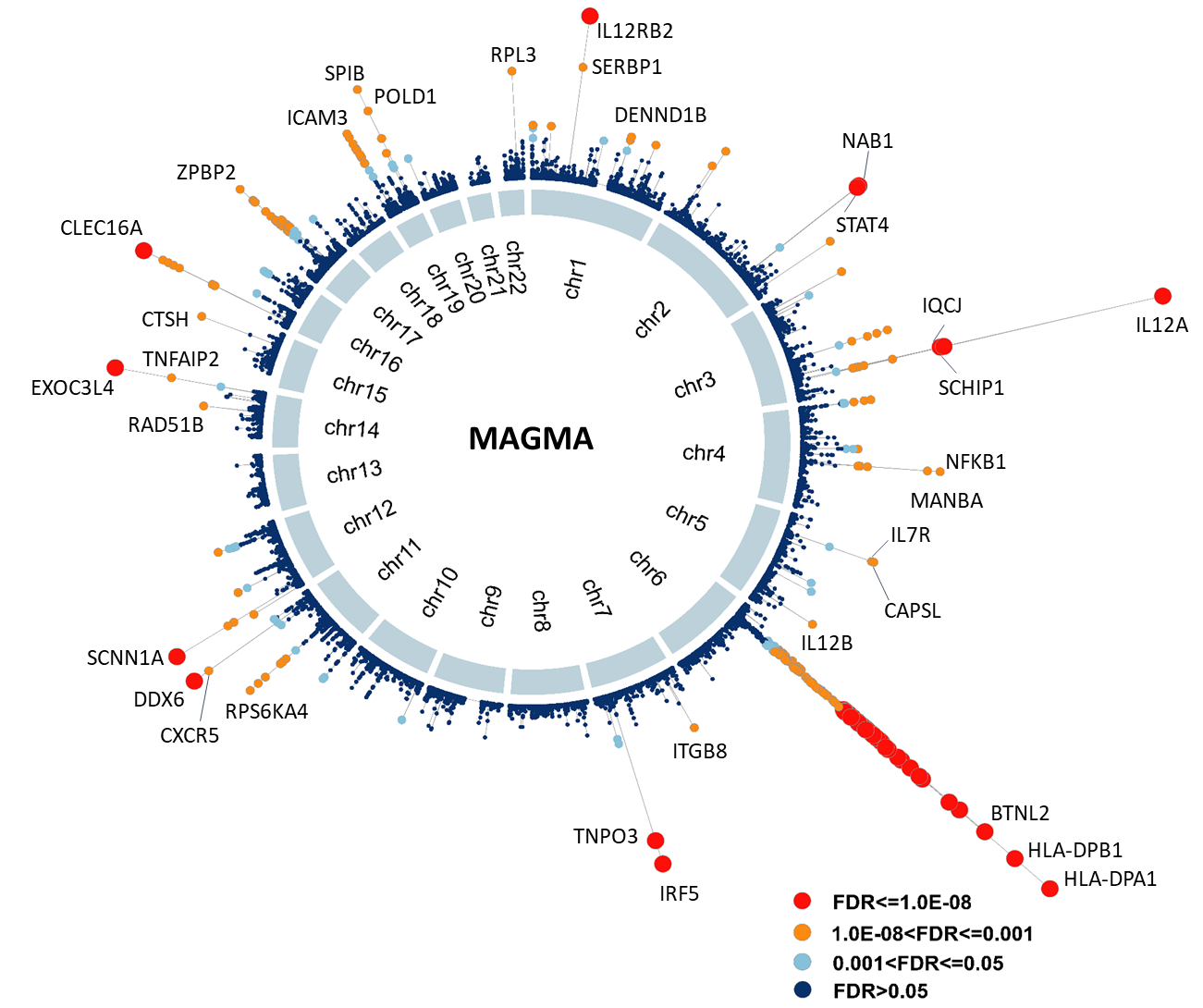
**

**Additional file Figure S2. MAGMA-based functional annotation analysis for GWAS summary data.** a) MAGMA-based pathway enrichment analysis based on GWAS summary data on PBC. b) Multidimensional scaling analysis of 41 significant pathways identified MAGMA-based pathway analysis. Note: Circular ring size represents the number of each enriched pathway. Color stands for the exponent function transformed *beta* value of each enriched pathway. Number of circular ring in the plot represents the pathway ID as shown in the **Additional file** Table S3.


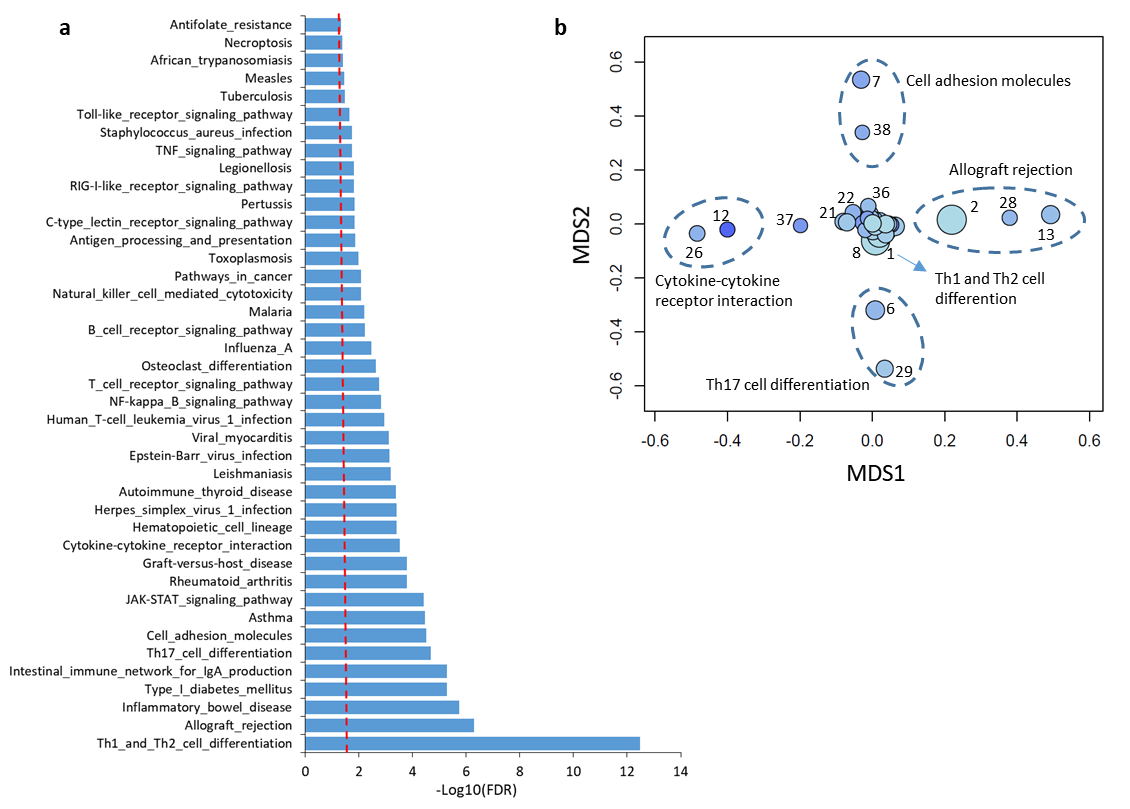


**Additional file Figure S3. Results of MAGMA gene-property analysis based on the web-access tool of FUMA.** Gray color means non-significant enrichment. Orange color stands for significant enrichment.


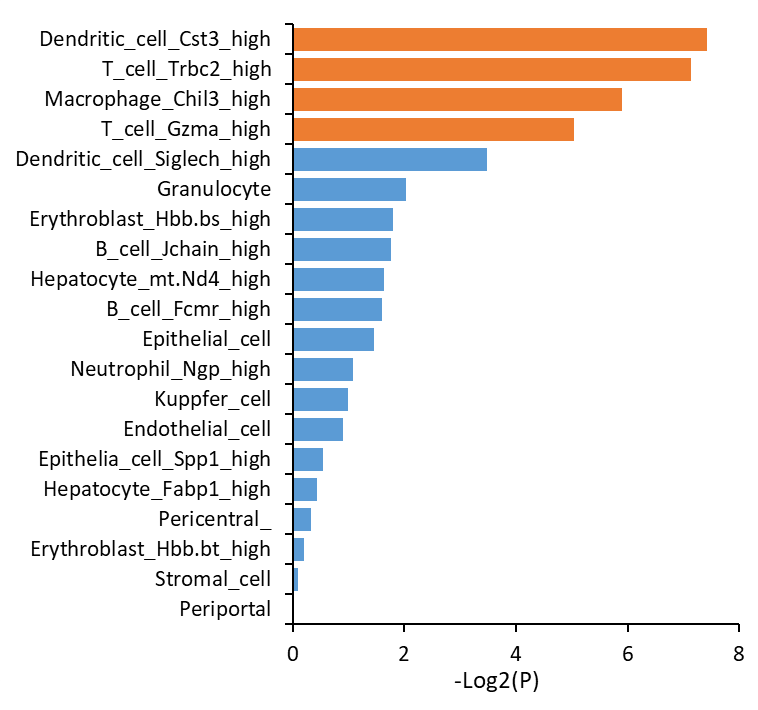


**Additional file Figure S4. Venn plot shows the overlap of results between MAGMA and S-MultiXcan analysis.** Note: There is a high overlap rate of 86.6% (232/268) between significant genes from S-MultiXcan analysis and that from MAGMA analysis.


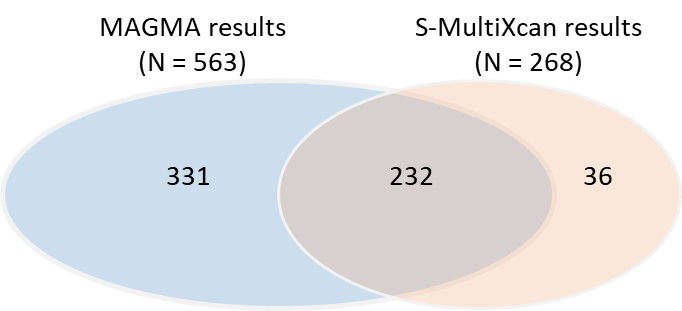


**Additional file Figure S5. Permutation analysis of 100,000 times for results from MAGMA and S-PrediXcan (liver and blood) compared with results from S-MultiXcan across multiple tissues.** a) for the comparison between MAGMA and S-MultiXcan (the observed number of overlapped genes is 232, permuted P < 1×10^-5^); b) for the comparison between S-PrediXcan on liver and S-MultiXcan (the observed number of overlapped genes is 64, permuted P < 1×10^-5^), and c) for the comparison between S-PrediXcan on blood and S-MultiXcan (the observed number of overlapped genes is 91, permuted P < 1×10^-5^). Note: the observed gene from each method was based on the significant threshold at FDR ≤ 0.05.


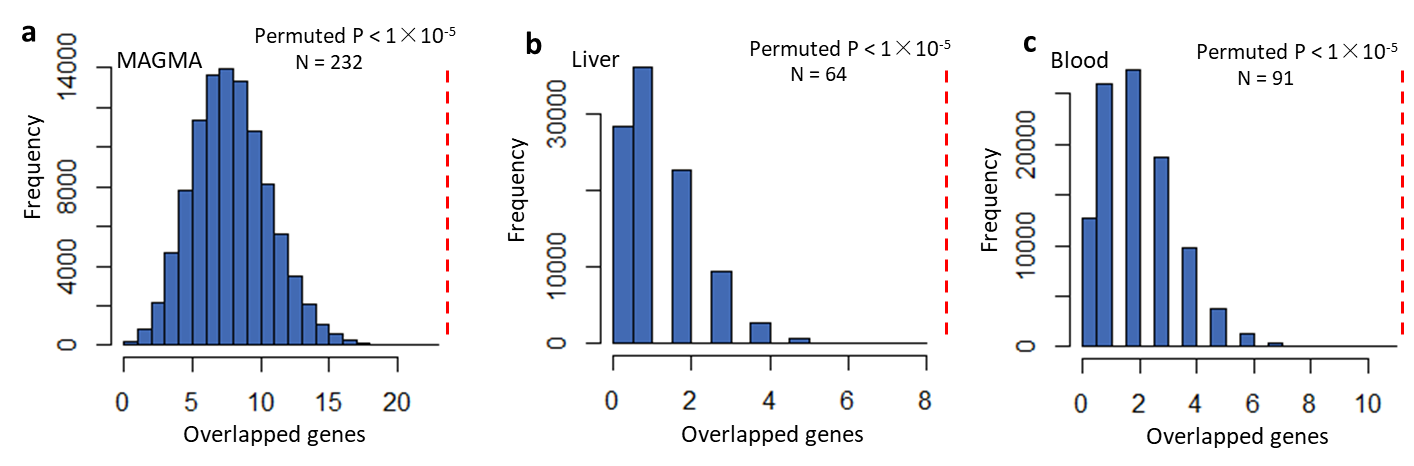


**Additional file Figure S6. Differential gene expression analysis of 29 risk genes based on bulk RNA profiles of liver tissue (Dataset of GSE159676).** a)-e) Violin plot showing the significantly differential expressions of genes in the dataset of GSE159676 between healthy controls and PBC patients.


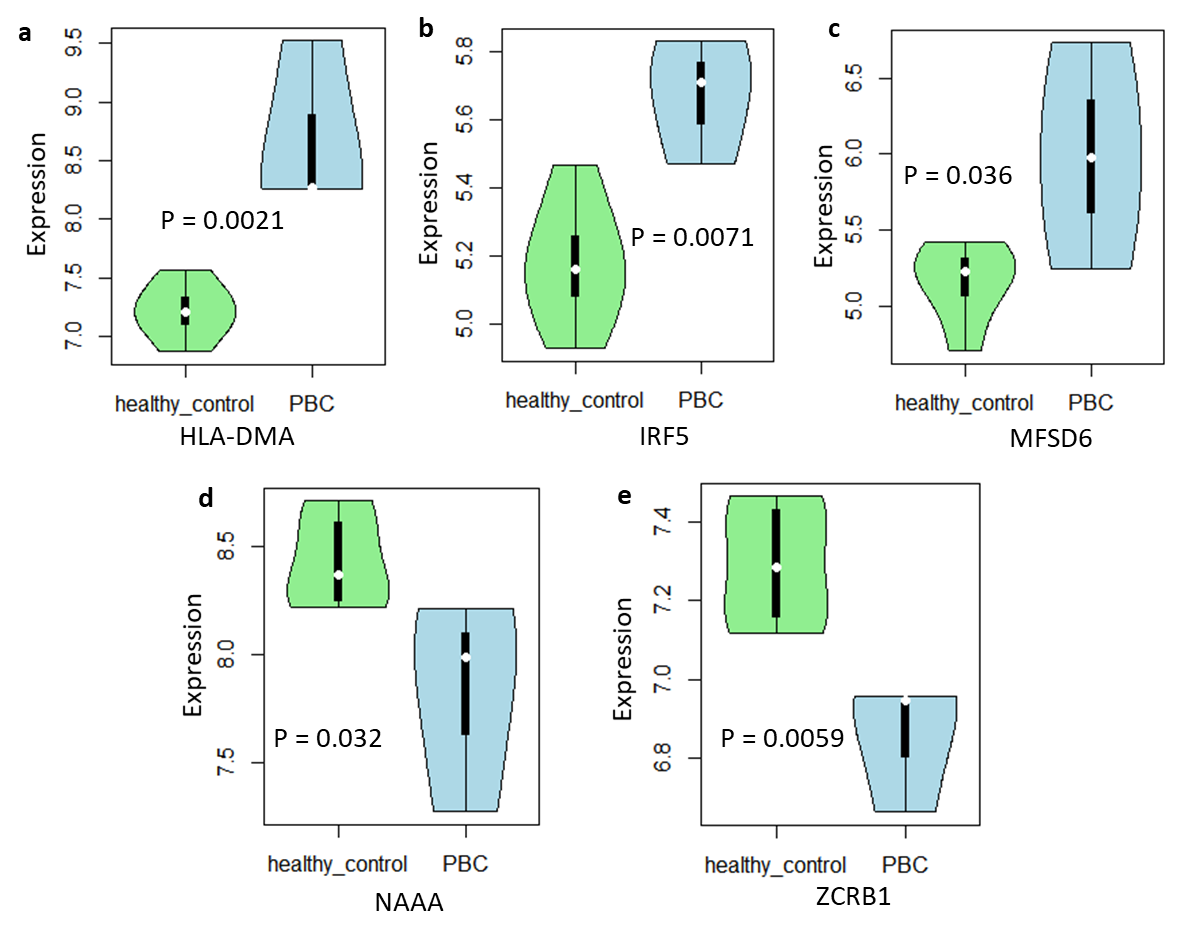


**Additional file Figure S7. Differential gene expression analysis of 29 risk genes based on bulk RNA profiles of blood tissue (Dataset of GSE119600).** a)-m) Violin plot showing the significantly differential expressions of genes in the dataset of GSE119600 between healthy controls and PBC patients.


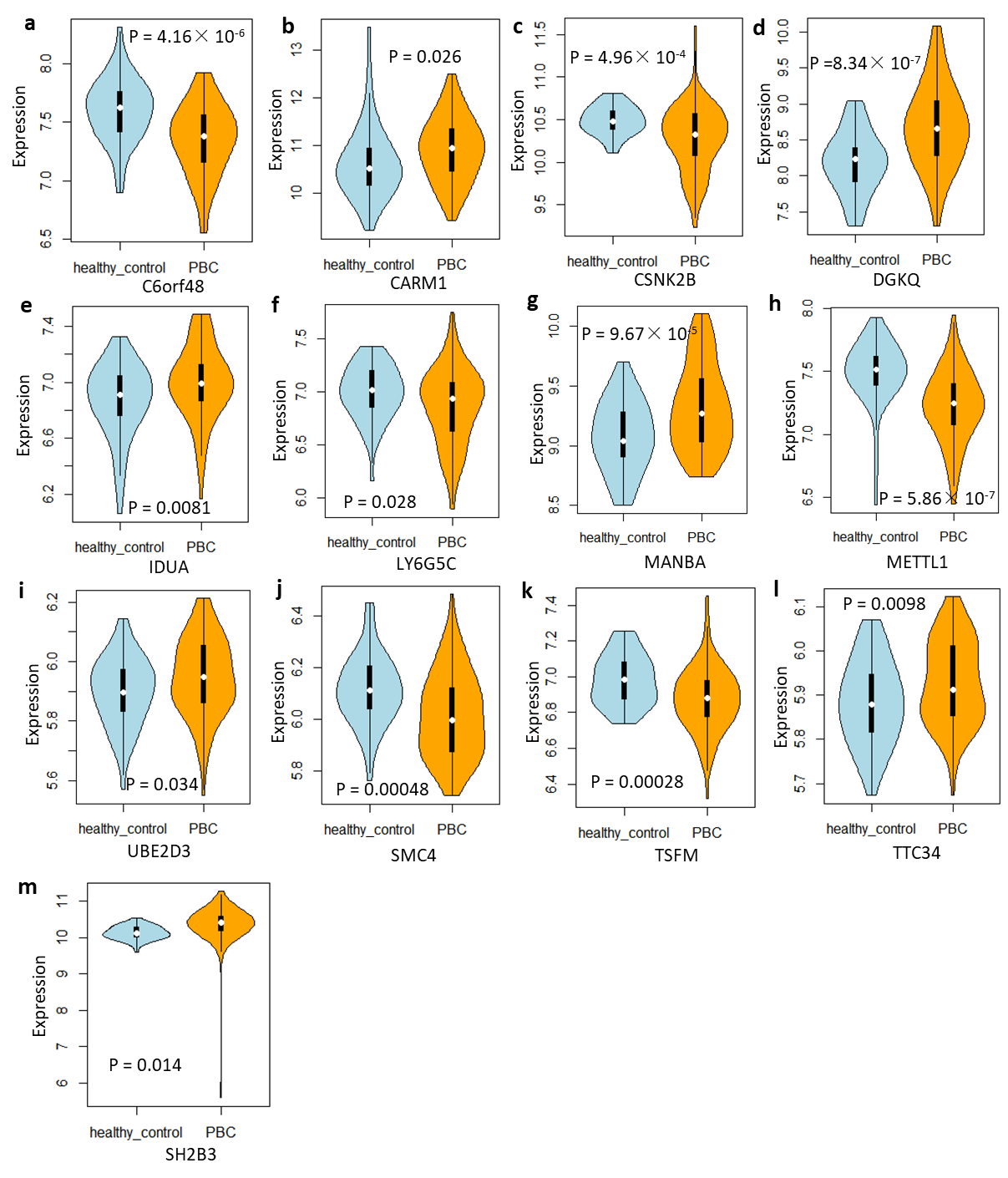


**Additional file Figure S8. Differential gene expression analysis of 29 risk genes based on bulk RNA profiles in CD4+T cells (Dataset of GSE93170).** a) Results of co-expression patterns among 29 genes in CD4+T cells between control group and PBC group. b)-i) Boxplot showing the significantly differential expression of genes in CD4+T cells between healthy control and PBC patients. b) CSNK2B, c) DDAH2, d) KPNA4, e) SOCS1, f) TSFM, g)SYNGR1, h) UBE2D3, i) ZCRB1.


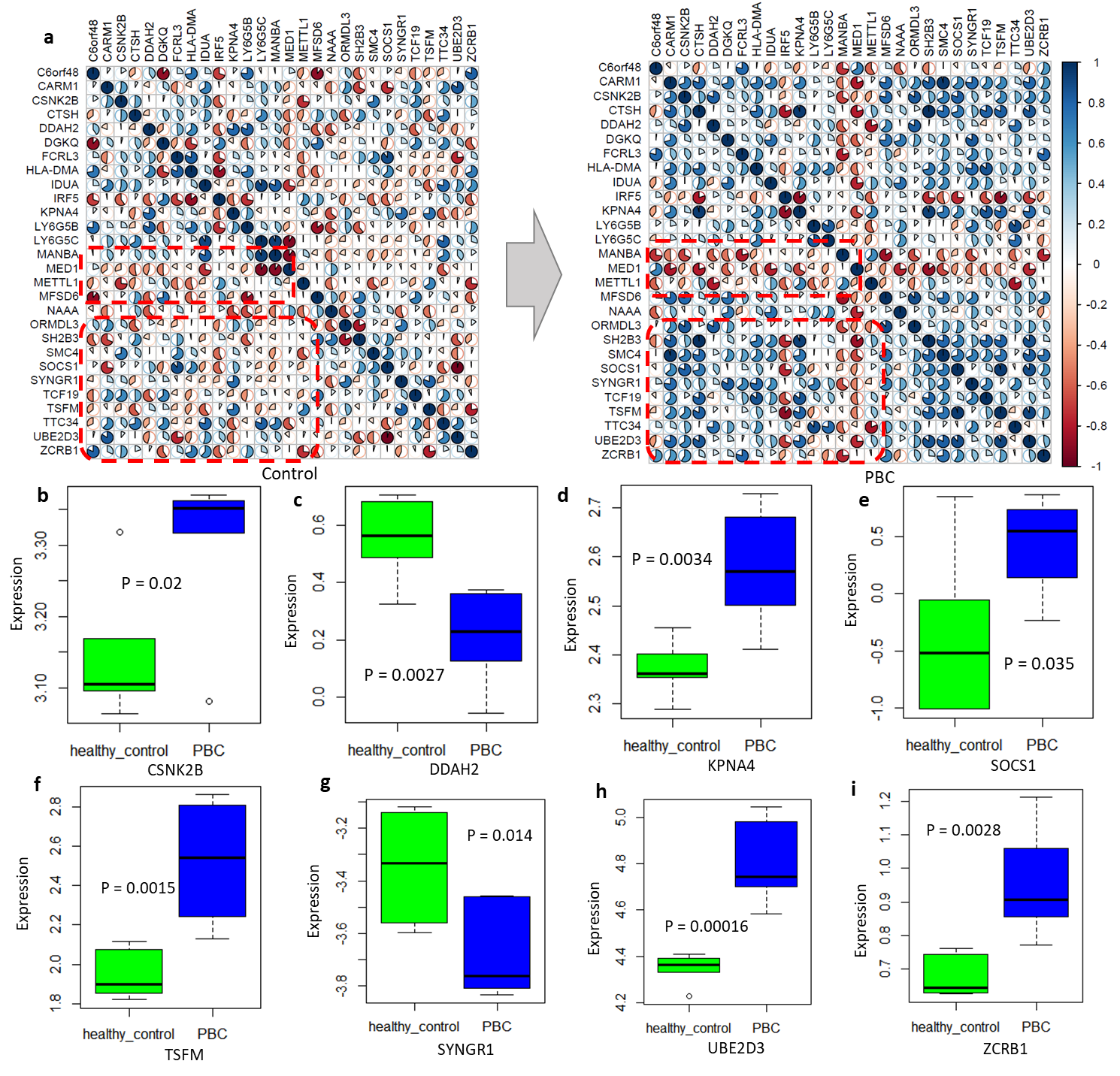


**Additional file Figure S9. Functional characterization of 29 risk associated with PBC**. a) Schematic diagram showing the literature mining and GWAS catalog searching for novel PBC-risk genes. b) Phenotype-based enrichment analysis based on the GLAD4U database for 29 risk genes.


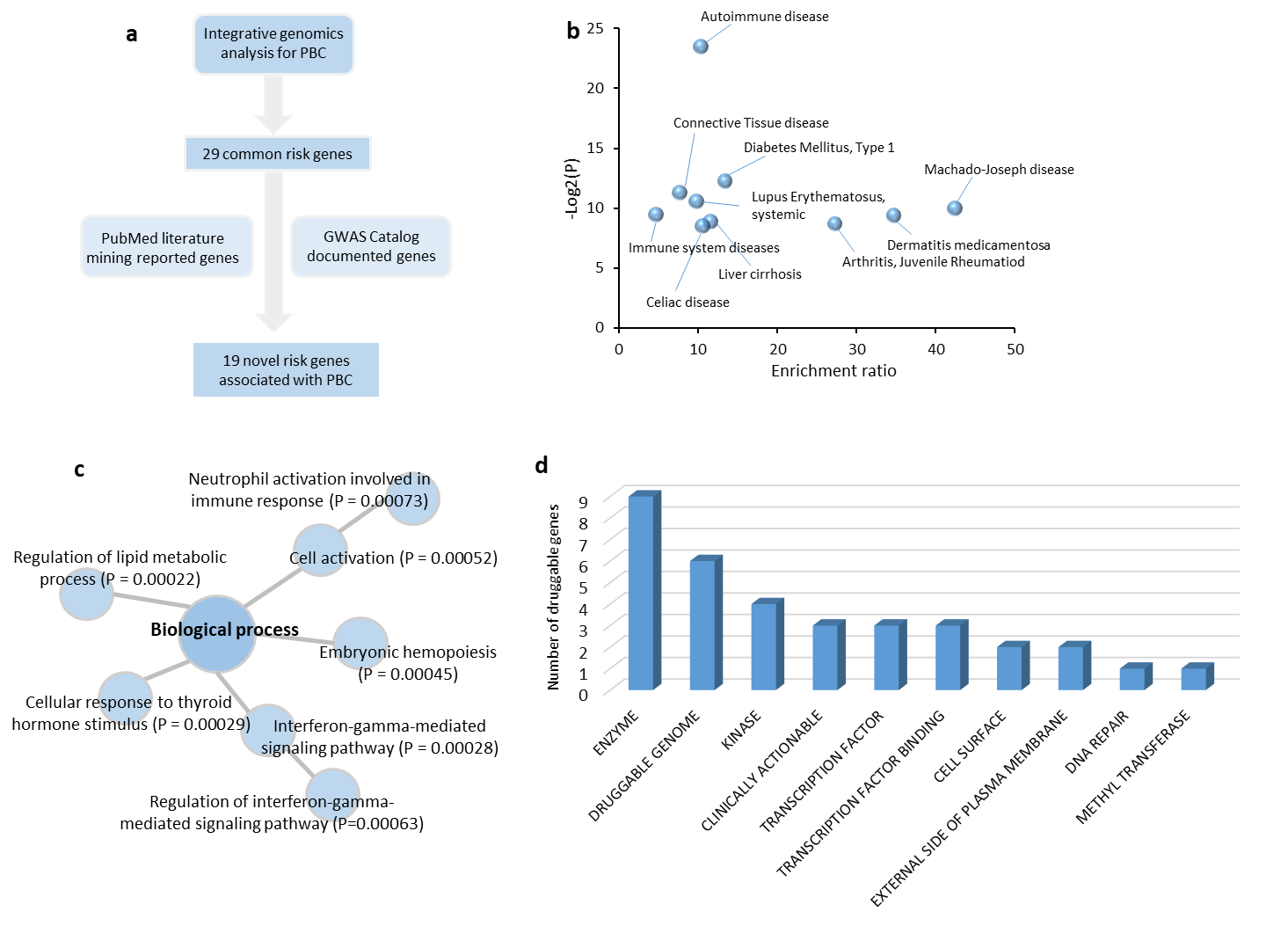


**Additional file Figure S10. Functional enrichment analysis of GO-term of biological process for 29 PBC-risk genes.**


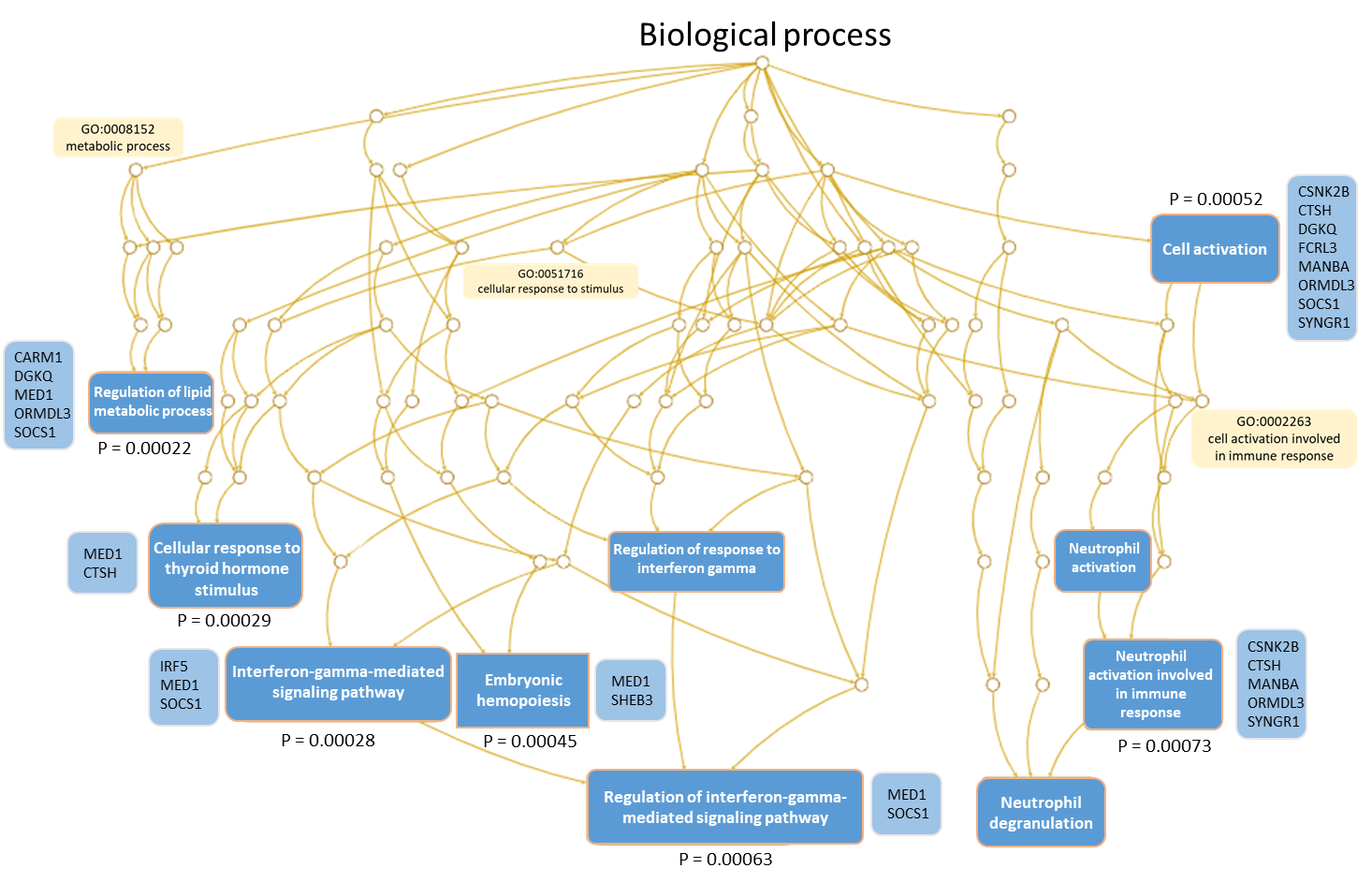


**Additional file Figure S11. Functional enrichment analysis of GO-term of cellular components for 29 PBC-risk genes.**


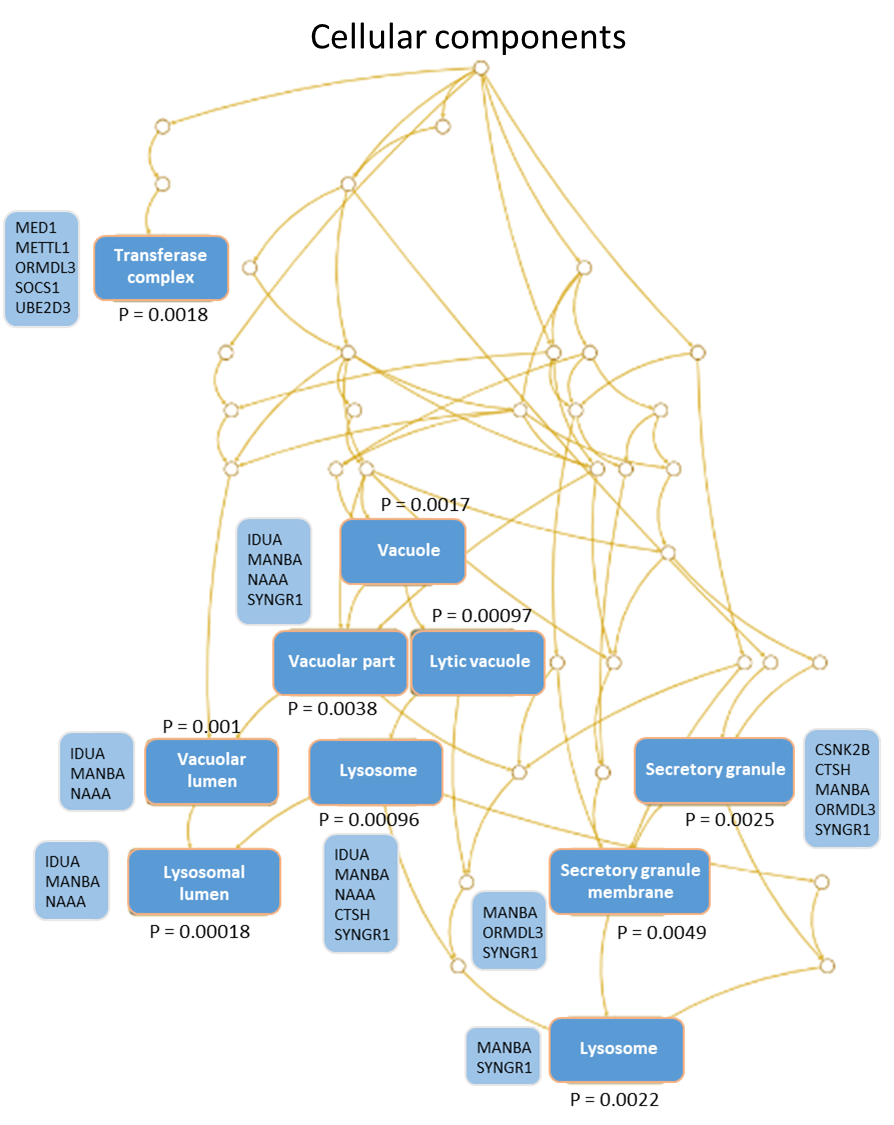


**Additional file Figure S12.** **Functional enrichment analysis of GO-term of molecular function for 29 PBC-risk genes.**


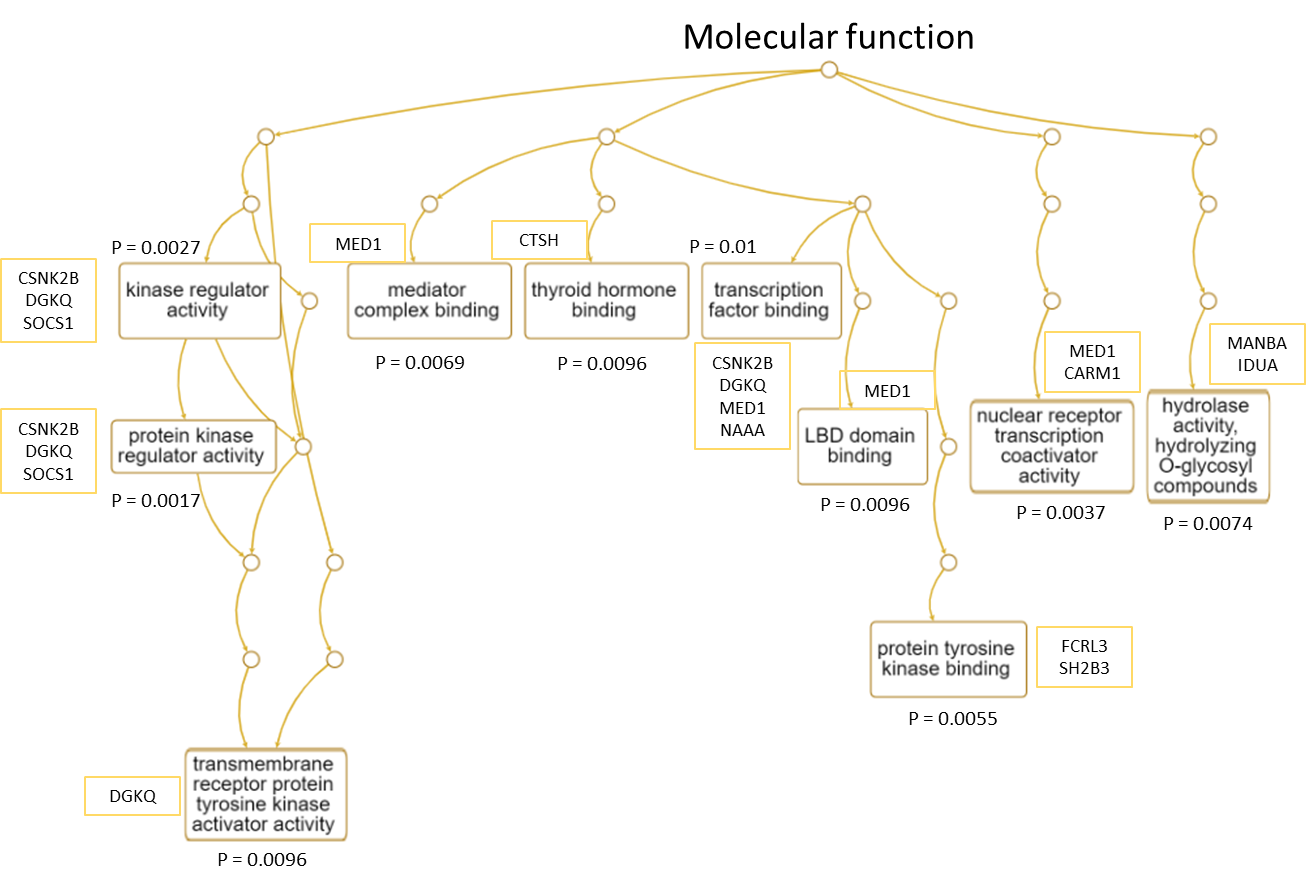


**Additional file Figure S13. Gene-drug interaction analysis for these identified PBC-associated risk genes.** a) Drug-gene interaction analysis showing 10 druggable gene categories. b) Drug-gene subnetwork. Note: Orange ring represents a specific gene, and blue triangle represents a given drug. The drug-gene interactions were plotted on the basis of the DGIdb database (<https://www.dgidb.org/>).

**
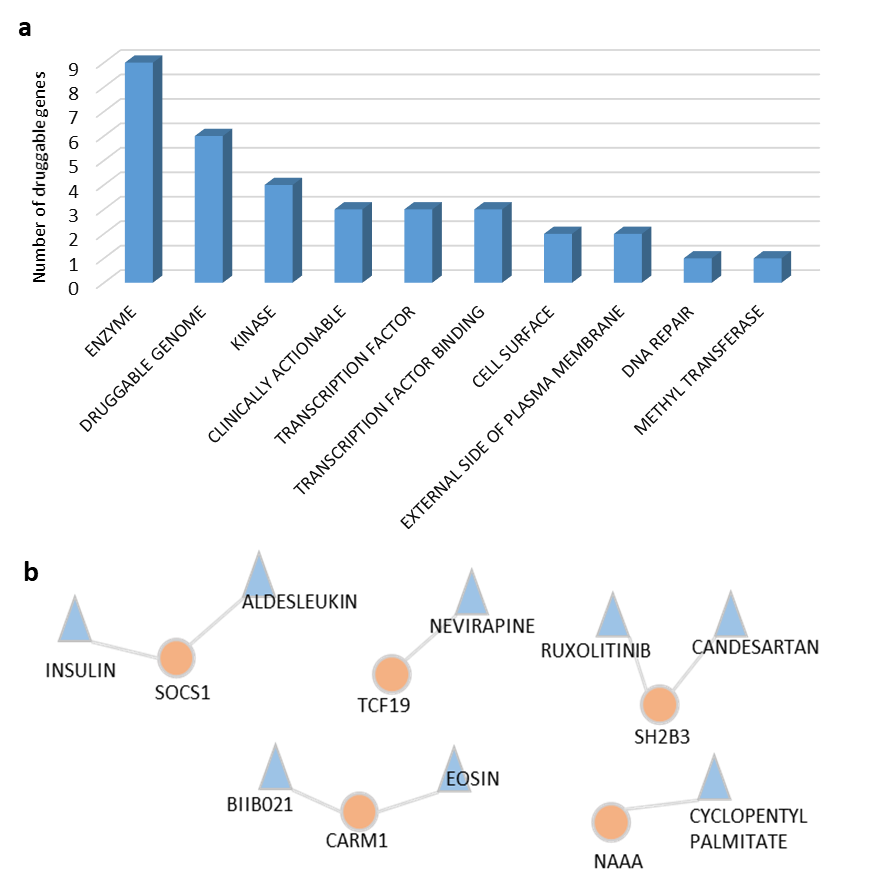
**

**Additional file Figure S14. Violin plot showing the difference in the expression level of *Ormdl3* between cholangitis-affected mice and healthy controls.** The mouse liver expression profiles on healthy sex- and age-matched mice (n=4) and mice suffering from cholangitis (n = 6) were downloaded from the Gene Expression Omnibus database (Accession number: GSE179993). Both healthy mouse and cholangitis-affected mouse lives were collected at day 35 post-cholangitis induction. The raw expression level sequenced using the Illumina NextSeq 500 (Mus musculus) was log transformed for normalization. Two-side Student’s t test was used for assessing statistical significance.


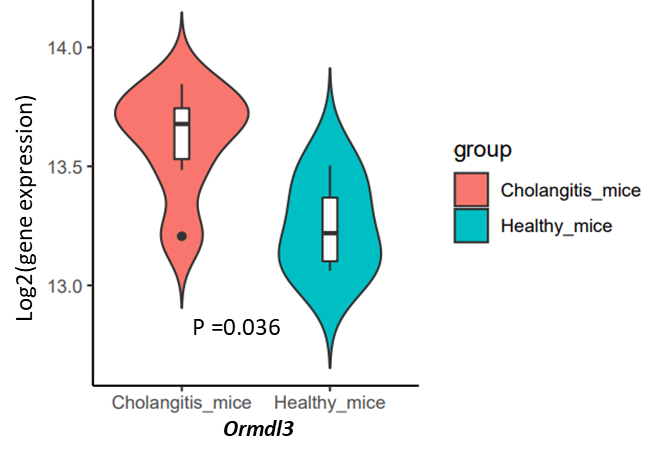


**Additional file Figure S15. The expression of ORMDL3 examined in PBC livers and paracarcinoma of intrahepatic metastasis by immunohistochemistry.** Note: Scale bar = 100 µm. The detailed information for this analysis, please refer to the Methods section.


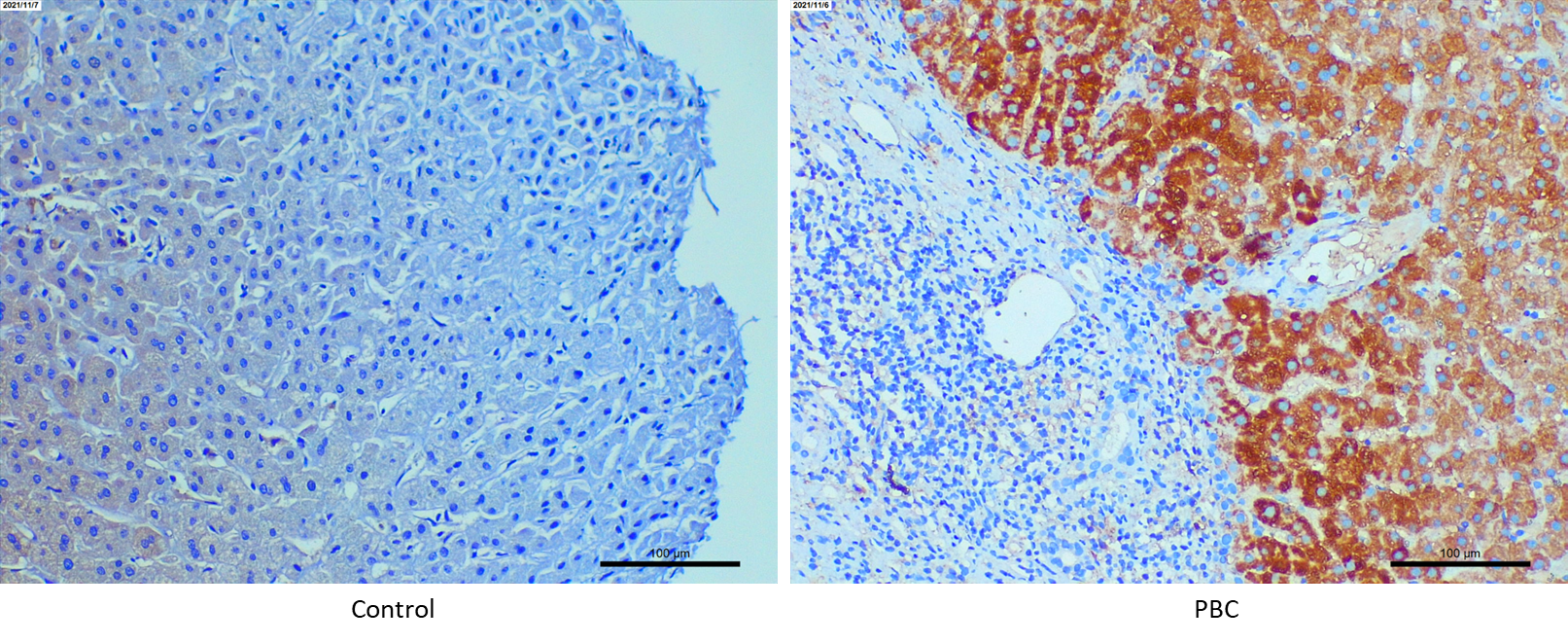


**Additional file Figure S16.** **Box plots showing the effects of different genotypes (CC, CT, and TT) of rs9303277 on the expression of *ORMDL3* among different immune cell types.** This dataset was based on the Database of Immune Cell Expression quantitative trait loci and Epigenomics (DICE, <https://dice-database.org/landing>)


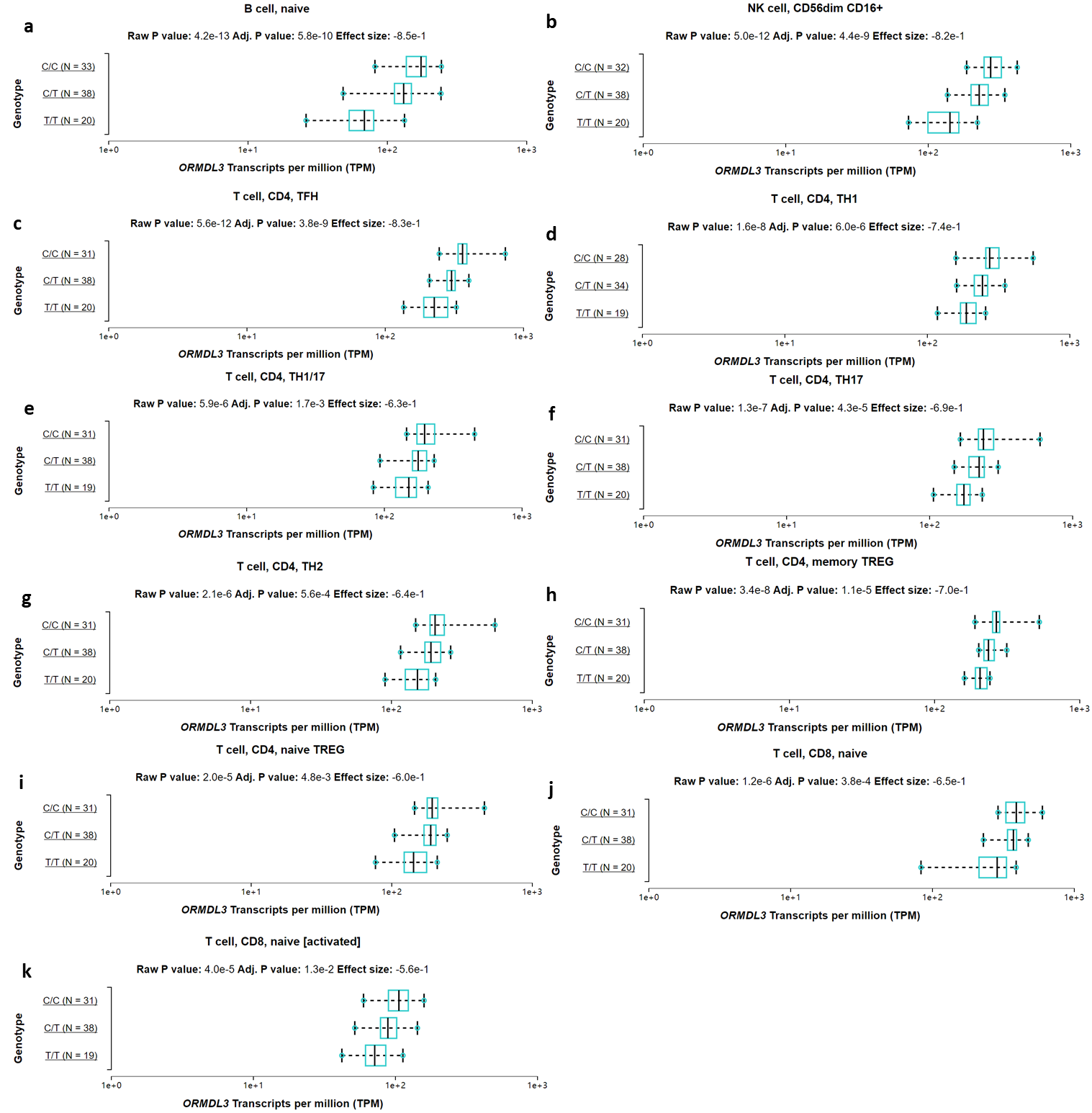


**Additional file Figure S17. Functional enrichment analysis of differential expressed genes between *ORMDL3*^+^ and *ORMDL3*^-^ cholangiocytes.** (a) 6 up-regulated DEGs based on the KEGG pathway resource. b)-d) GO enrichment analyses of 71 down-regulated DEGs according to three categories: (b) biological process, (c) cellular component, and (d) molecular function. FDR < 0.05 represents a significantly enriched pathway or GO-term.


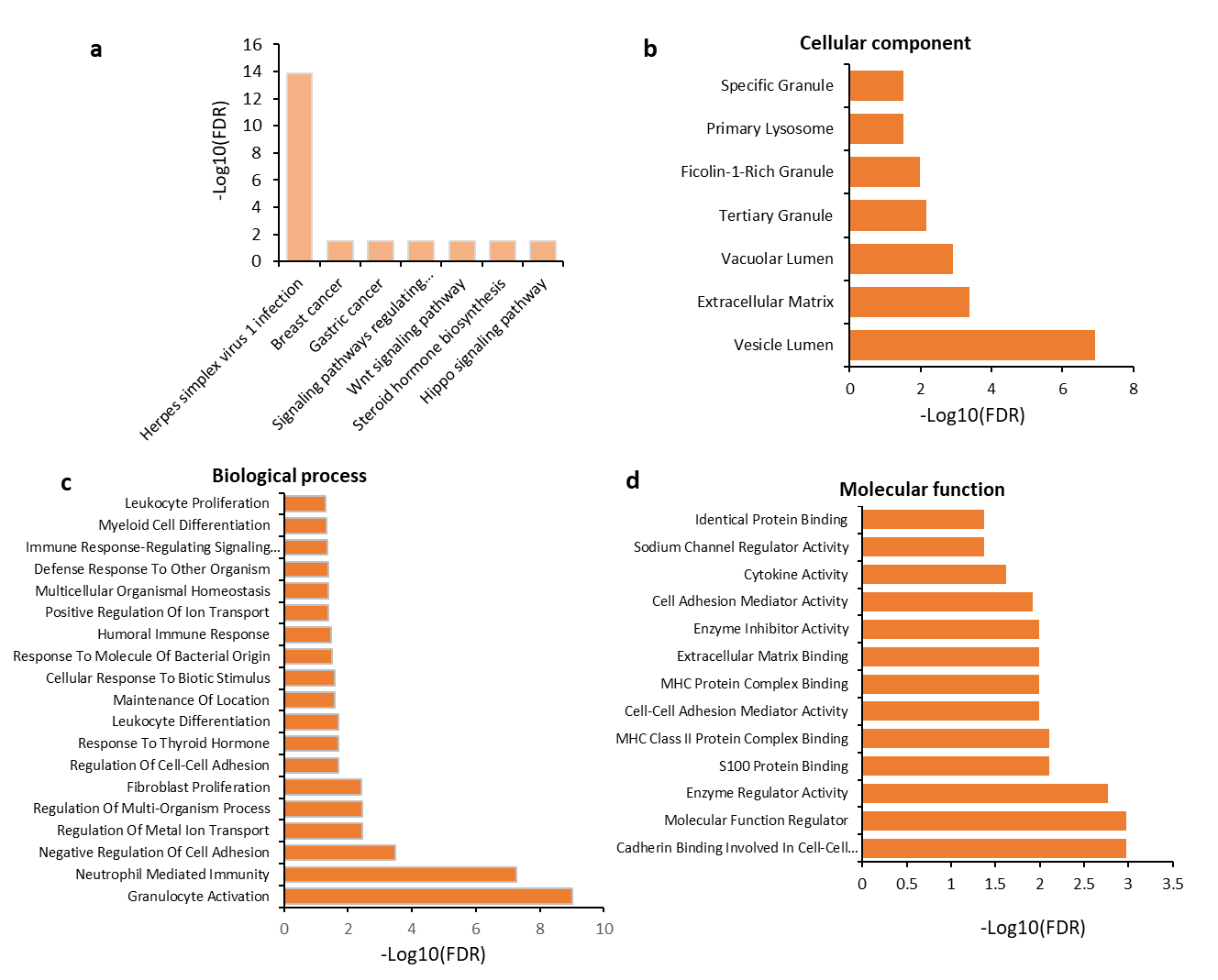


### Additional file Figure S18. Identify signals contributing most to outgoing (sources) or incoming (targets) signaling of cell populations in liver tissues (GSE115469). (a) Outgoing signaling patterns among 13 cell types, (b) Incoming signaling patterns among cell types. The color of heatmap represents the relative strength of cellular interactions.

**
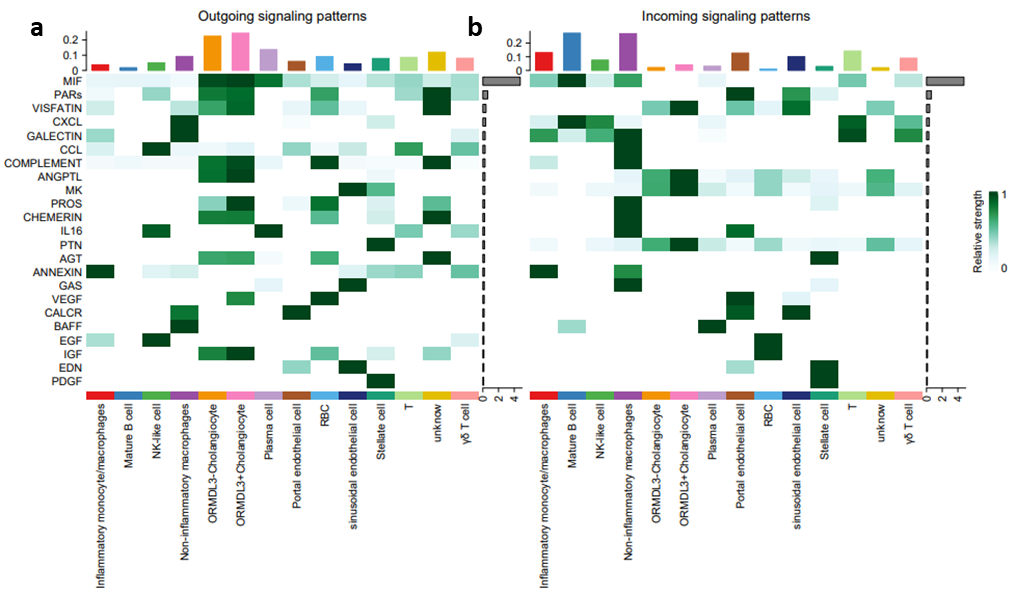
**

**Additional file Figure S19. The aggregated cell-to-cell communication network among cell populations in liver tissues (GSE115469).** The width represents the number of cellular interactions.


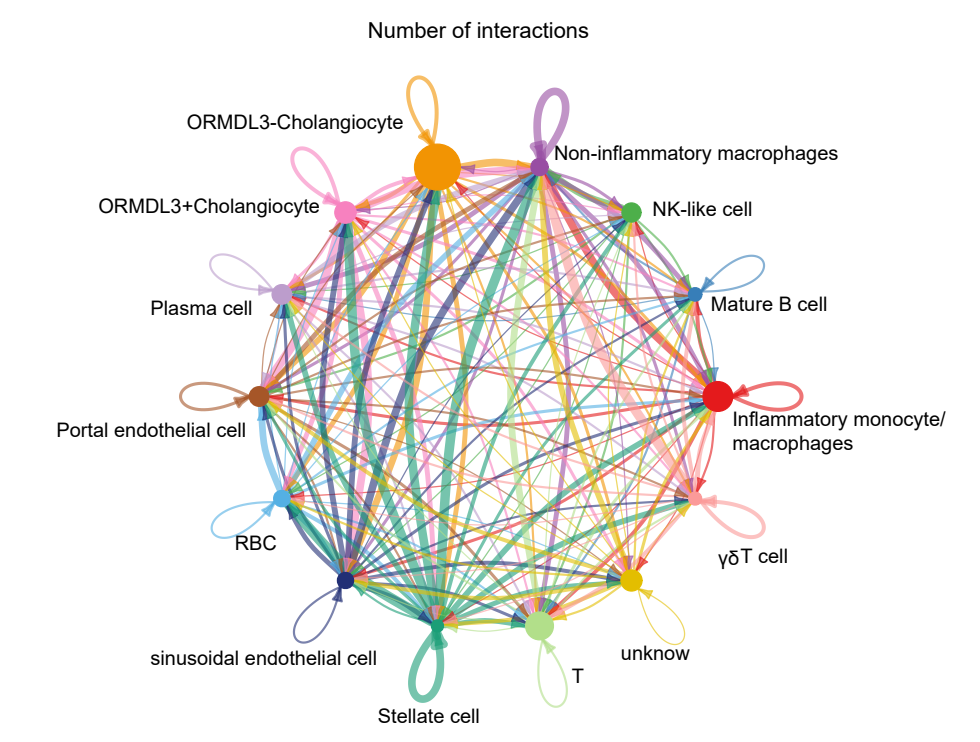


**Additional file Figure S20. The cellular communications of *ORMDL3^-^* Cholangiocyte with other cell populations in liver tissues (GSE115469).** The width represents the number of cellular interactions, and color represents each cell type.


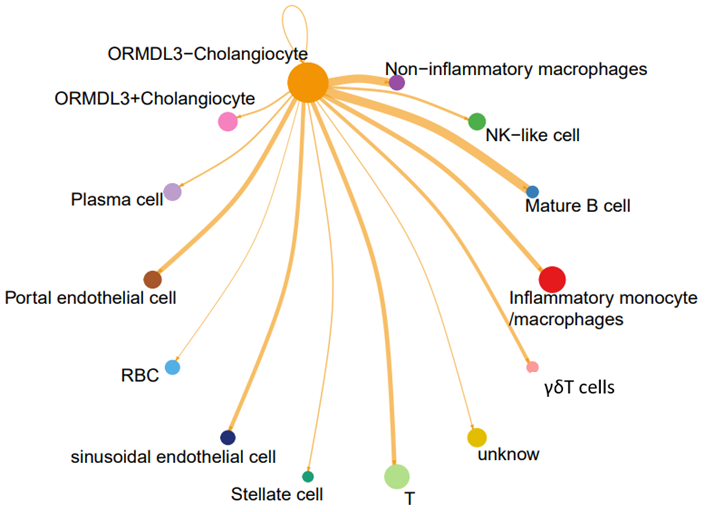


**Additional file Figure S21. Dot plots showing that predicted cellular interactions of *ORMDL3^-^* Cholangiocyte with other cell populations in liver tissues (GSE115469).** (a) *ORMDL3^-^* Cholangiocyte as a source (outgoing) communicating with other cells, (b) *ORMDL3^-^* Cholangiocyte as a target (incoming) communicating with other cells. The circular size of represents the statistical significance of each ligand-receptor pair, and color represents the communication probability.


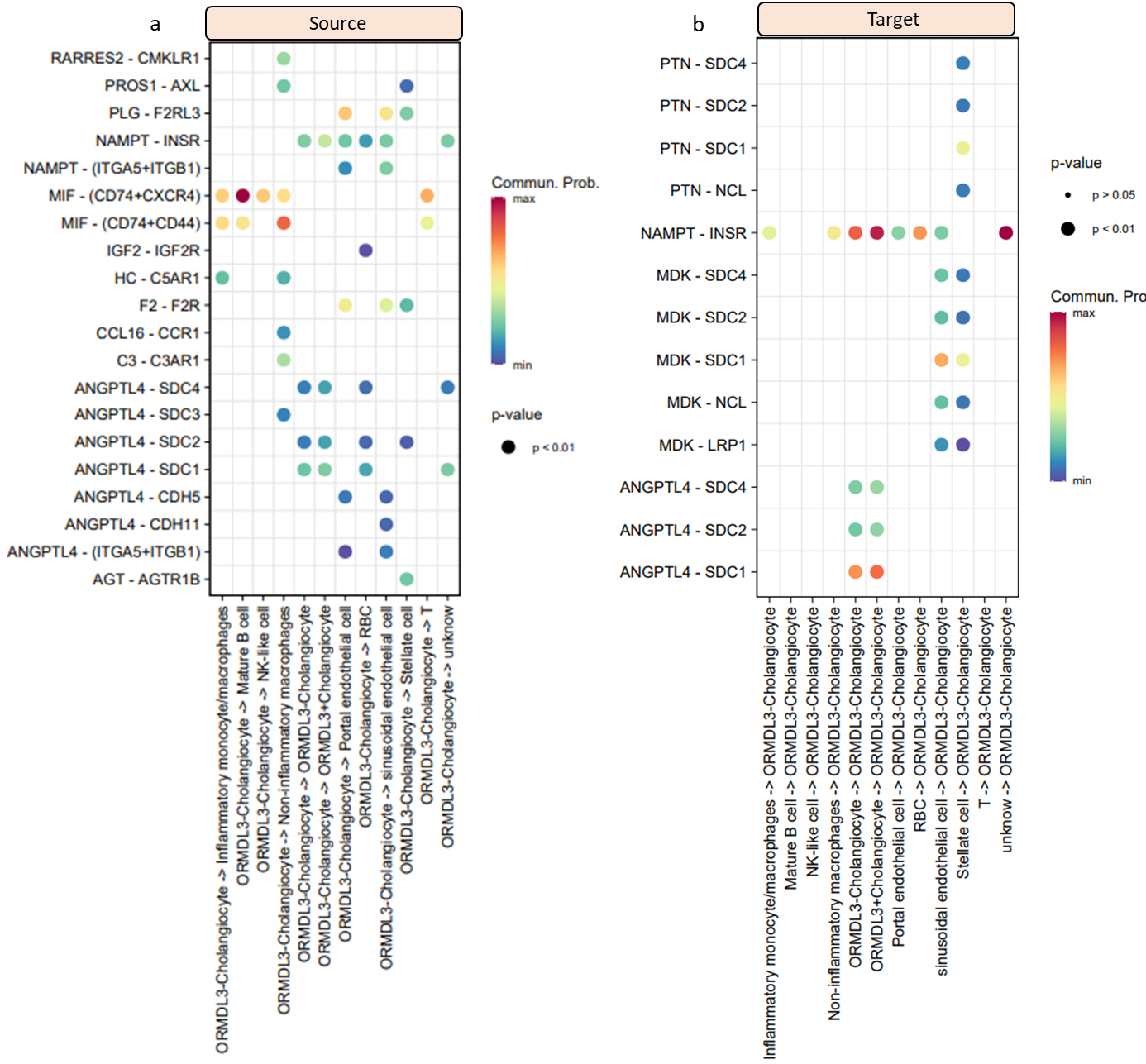


**Additional file Figure S22. The inferred incoming (target) communication patterns of cell populations in liver tissue (GSE115469).** (a) Five incoming communications patterns of 13 cell populations, which shows the correspondence between the inferred latent patterns and cell groups, as well as signaling pathways, (b) The contribution of signaling pathways among the five incoming communications patterns to liver and immune cell types.


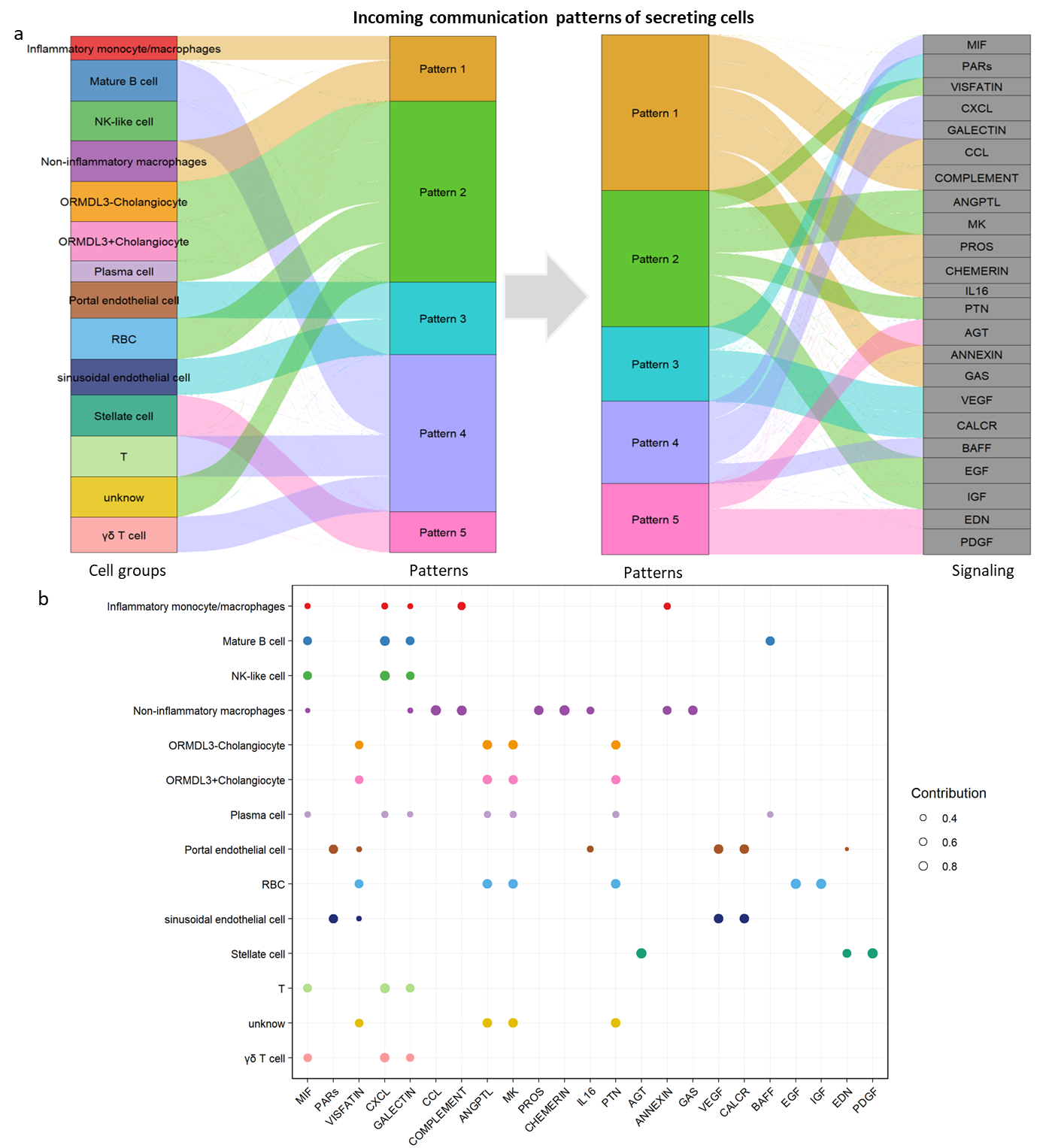


**Additional file Figure S23. Relative contribution of each ligand-receptor pair to that of VEGF signaling pathway.** The relative contribution is the ratio of the total communication probability of the inferred network of each ligand-receptor pair to that of VEGF signaling pathway.


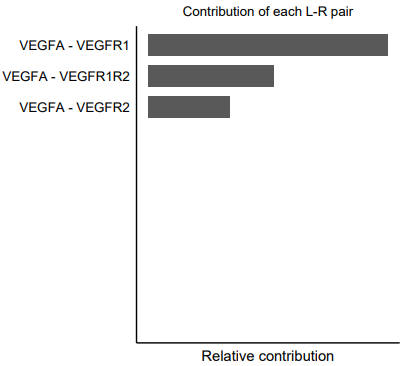

Supplement: Supplementary file 1 — Additional File 1: Figure S1. Circus plot showing the results of gene-level genetic association analysis of GWAS summary statistics on PBC. Figure S2. MAGMA-based functional annotation analysis for GWAS summary data. a) MAGMA-based pathway enrichment analysis based on GWAS summary data on PBC. b) Multidimensional scaling analysis of 41 significant pathways identified MAGMA-based pathway analysis. Figure S4. Results of MAGMA gene-property analysis based on the web-access tool of FUMA. Figure S2. Venn plot shows the overlap of results between MAGMA and S-MultiXcan analysis. Figure S5. Permutation analysis of 100,000 times for results from MAGMA and S-PrediXcan (liver and blood) compared with results from S-MultiXcan across multiple tissues. Figure S6. Differential gene expression analysis of 29 risk genes based on bulk RNA profiles of liver tissue (Dataset of GSE159676). Figure S7. Differential gene expression analysis of 29 risk genes based on bulk RNA profiles of blood tissue (Dataset of GSE119600). Figure S8. Differential gene expression analysis of 29 risk genes based on bulk RNA profiles in CD4+T cells (Dataset of GSE93170). Figure S9. Functional characterization of 29 risk associated with PBC. a) Schematic diagram showing the literature mining and GWAS catalog searching for novel PBC-risk genes. b) Phenotype-based enrichment analysis based on the GLAD4U database for 29 risk genes. Figure S10. Functional enrichment analysis of GO-term of biological process for 29 PBC-risk genes. Figure S11. Functional enrichment analysis of GO-term of cellular components for 29 PBC-risk genes. Figure S12. Functional enrichment analysis of GO-term of molecular function for 29 PBC-risk genes. Figure S13. Gene-drug interaction analysis for these identified PBC-associated risk genes. a) Drug-gene interaction analysis showing 10 druggable gene categories. b) Drug-gene subnetwork. Figure S14. Violin plot showing the difference in the expression level of Ormdl3 between cholangitis-affec [file 12951_2021_1154_MOESM1_ESM.docx]
